# Transcriptional regulatory network analysis during epithelial-mesenchymal transformation of retinal pigment epithelium

**Published:** 2008-08-04

**Authors:** Craig H. Pratt, Rajanikanth Vadigepalli, Praveen Chakravarthula, Gregory E. Gonye, Nancy J. Philp, Gerald B. Grunwald

**Affiliations:** Department of Pathology, Anatomy and Cell Biology, Thomas Jefferson University, Philadelphia, PA

## Abstract

**Purpose:**

Phenotypic transformation of retinal pigment epithelial (RPE) cells contributes to the onset and progression of ocular proliferative disorders such as proliferative vitreoretinopathy (PVR). The formation of epiretinal membranes in PVR may involve an epithelial-mesenchymal transformation (EMT) of RPE cells as part of an aberrant wound healing response. While the underlying mechanism remains unclear, this likely involves changes in RPE cell gene expression under the control of specific transcription factors (TFs). Thus, the purpose of the present study was to identify TFs that may play a role in this process.

**Methods:**

Regulatory regions of genes that are differentially regulated during phenotypic transformation of ARPE-19 cells, a human RPE cell line, were subjected to computational analysis using the promoter analysis and interaction network toolset (PAINT). The PAINT analysis was used to identify transcription response elements (TREs) statistically overrepresented in the promoter and first intron regions of two reciprocally regulated RPE gene clusters, across four species including the human genome. These TREs were then used to construct transcriptional regulatory network models of the two RPE gene clusters. The validity of these models was then tested using RT–PCR to detect differential expression of the corresponding TF mRNAs during RPE differentiation in both undifferentiated and differentiated ARPE-19 and primary chicken RPE cell cultures.

**Results:**

The computational analysis resulted in the successful identification of specific transcription response elements (TREs) and their cognate TFs that are candidates for serving as nodes in a transcriptional regulatory network regulating EMT in RPE cells. The models predicted TFs whose differential expression during RPE EMT was successfully verified by reverse transcriptase polymerase chain reaction (RT–PCR) analysis, including Oct-1, hepatocyte nuclear factor 1 (HNF-1), similar to mothers against decapentaplegic 3 (SMAD3), transcription factor E (TFE), core binding factor, erythroid transcription factor-1 (GATA-1), interferon regulatory factor-1 (IRF), natural killer homeobox 3A (NKX3A), Sterol regulatory element binding protein-1 (SREBP-1), and lymphocyte enhancer factor-1 (LEF-1).

**Conclusions:**

These studies successfully applied computational modeling and biochemical verification to identify biologically relevant transcription factors that are likely to regulate RPE cell phenotype and pathological changes in RPE in response to diseases or trauma. These TFs may provide potential therapeutic targets for the prevention and treatment of ocular proliferative disorders such as PVR.

## Introduction

The retinal pigment epithelium (RPE) is a monolayer of hexagonally packed, highly pigmented, polarized cells located on the posterior wall of the eye, whose apical membranes are intimately associated with the outer segments of photoreceptor cells of the neural retina (NR). The RPE forms the outer blood-retinal barrier and carries out important physiologic and protective functions necessary for visual processing in rod and cone cells, such as retinoid metabolism, phagocytosis of discarded rod and cone outer segments, absorption of stray light to preserve visual acuity, control of water and ion flow between the neural retina and choroid, and protection of the neural retina from oxidative damage [1–3]. These functions require maintenance of intimate association of the RPE with the NR, which if disrupted leads to severe ocular pathologies. In situ, the RPE is both non-proliferative and non-migratory, yet these cells have been shown to exhibit a high degree of plasticity in vitro. The plasticity in function and phenotype of the RPE can be recapitulated in vivo when damage occurs to the retina in the form of a retinal detachment or tear. Therefore retinal detachments and tears require surgical repair of the RPE/NR interaction [4]. However, as many as 10% of all rhegmatogenous retinal detachments, in which a retinal tear occurs, result in failure due to the occurrence of proliferative vitreoretinopathy (PVR).

PVR is characterized by the formation of epiretinal membranes, comprised in part of dedifferentiated RPE cells that undergo epithelial-mesenchymal transformation (EMT) and contribute to this fibroplastic response [2,5,6]. While the cause of PVR remains unknown, one aspect of this disease includes changes in the expression of a variety of genes that regulate RPE cell phenotype. Identification of the transcription factors that maintain RPE cells in a differentiated non-proliferative and non-migratory state could provide potential therapeutic targets. Studies of EMT in a variety of cell types in development and disease have begun to identity such factors [7-9]. More recently, high-throughput technology such as microarray analysis has identified changes in gene expression in RPE cells undergoing EMT in vitro, including genes involved in DNA synthesis and repair, cell cycle, intracellular signaling, and cell adhesion [10,11]. However, the mechanisms by which these many changes are controlled during EMT, including the transcriptional regulators that may coordinate this process, remain to be elucidated.

The purpose of the present study was to identify transcription factors that regulate EMT in RPE cells. Genes were identified that are upregulated or down-regulated during RPE EMT, and the genomics tool Promoter Analysis and Interaction Network Toolset (PAINT v 3.3) [12] was then used to generate models of the promoter regions of these genes including predictions of those TFs that regulate their expression. We then tested the validity of these models using RT–PCR to analyze expression of the TFs in differentiated and undifferentiated RPE cells and indeed identified several TFs that are differentially expressed between these two RPE cell states. The results of these studies indicate that a combination of computational and biochemical approaches can be successfully applied to analyze these complex events.

## Methods

### Computational analysis of gene regulatory regions

Identification and analysis of the regulatory regions of genes expressed in RPE cells was performed using the Promoter Analysis and Interaction Network Toolset version 3.3 (PAINT) program [12]. The target gene set analyzed comprised sixty genes differentially expressed during EMT of RPE cells, selected from previous literature reports and additional genes under study in our laboratory [10,11] (Table 1). This set was divided into two subsets representing those preferentially expressed in the undifferentiated or differentiated state, and further refined by functional gene ontology [13]. Homologous genes in humans, mice, rats, and chickens were identified using the Ensembl database and the Ensembl gene ID for each was used in the PAINT program [14,15]. For each gene, PAINT identified the putative transcription start site (TSS) and subsequent promoter analysis to identify transcription response elements (TREs) was performed to 5,000 bp upstream of the TSS, with exclusion of complimentary strand analysis and 1.0 core similarity threshold [12]. In addition, sequence data corresponding to the first intron of each gene was retrieved from Ensembl and also entered into PAINT, as FASTA formatted sequences, for further analysis. TREs within both the promoters as well as first introns were identified using the MATCH/TRANSFAC database [16]. These TREs were entered into the Feasibility Network Builder module of PAINT (FeasNet Builder), which constructed a candidate interaction matrix (CIM), a graphic representation of the occurrence of these TREs within the gene set. Enrichment analysis was performed using PAINT to compute the Fisher's exact test p-values indicating relative over-representation of TREs within the selected gene set as compared to the larger background gene set, the 588 genes present on the Human Atlas Array (Cat. No. 7740–1; Clontech, Palo Alto, CA) used to originally identify the set of sixty differentially regulated RPE genes [10,11]. In each of the analyses, multiple testing correction was applied using a false discovery rate (FDR) estimate [17]. In all cases, the multiple testing corrections were not informative as they did not result in any over-represented TREs, since the FDR was consistently above 90% for all TREs. Therefore, we followed a discovery approach and chose a threshold of p<0.1 on the Fisher's exact test p-value to identify those TREs to be included in further filtering as described below. Models of RPE gene interaction networks, based upon results of the FeaseNet analysis, were graphically generated using GraphViz [18].

**Table 1:** Gene ontology list for target sequences used in PAINT analysis

**Gene Ontology**	**Gene name**	**Cluster**	**Gene Ontology**	**Gene name**	**Cluster**
			Growth factor binding	IGFBP-1	Undifferentiated
Cell cycle effectors	CDC25A	Undifferentiated		IGFBP-3	Undifferentiated
	Cyclin H	Undifferentiated		EMAP II	Undifferentiated
	Cdc2-related protein kinase	Undifferentiated		IL-13	Undifferentiated
	G(1)/G(S)/G(2) beta2	Undifferentiated			
			Cell death	Caspase 4	Undifferentiated
Cell adhesion	I-CAM-1	Undifferentiated		ICE LAP3	Undifferentiated
	V-CAM-1	Undifferentiated			
	E-selectin	Undifferentiated	Cell organization	alpha-SMA	Undifferentiated
	Integrin alpha4	Undifferentiated		ZO1	Differentiated
	Integrin beta5	Undifferentiated		Thymosin beta10	Differentiated
	N-cadherin	Undifferentiated			
	Fibronectin	Differentiated	Signal transduction	STK-2	Undifferentiated
	CD44 antigen	Differentiated		CAK	Undifferentiated
	Integrin alpha5	Differentiated		MAPK3	Undifferentiated
	Integrin beta4	Differentiated		PDGF-B	Undifferentiated
	R-cadherin	Differentiated		CD33 antigen	Undifferentiated
				RGS19IP1	Undifferentiated
Cell metabolism	CLK-1	Undifferentiated		FADK2	Undifferentiated
	STK-1	Undifferentiated		Tyk2	Undifferentiated
	Tyrosinase	Differentiated		RAB5A	Undifferentiated
	PEDF	Differentiated		PKCgamma17	Undifferentiated
	RPE65	Differentiated		IGF-1	Undifferentiated
	TYRP2	Differentiated		VEGF	Undifferentiated
				MCP-1	Undifferentiated
Ion binding	CAM IV	Undifferentiated		Neuromodulin	Undifferentiated
	SPARC/Osteonectin	Differentiated		FADK2	Undifferentiated
				PAK-C alpha	Undifferentiated
Transcription factors	CRE BP-1	Undifferentiated		FGFR-1	Differentiated
	TFAP 2	Undifferentiated		FGFR-3	Differentiated
				HDGF	Differentiated
Proliferation/differentiation	STAT6	Undifferentiated			
	GDF-1	Undifferentiated	Inter/intra cellular transport	MAL	Undifferentiated
				MCT-4	Undifferentiated
				MCT-3	Differentiated
				Bestrophin	Differentiated

The three columns indicate the gene name, gene ontology cluster assignment, and inclusion into either the differentiated or undifferentiated retinal pigment epithelium expression cluster. The genes were organized within the ontology clusters according to The Gene Ontology Consortium (2000).

### Generation of models for gene regulatory regions and selection of targets for biochemical analysis

Global regulatory models for gene sets coordinately expressed in RPE cells, as well as for individual genes differentially expressed in differentiated or undifferentiated RPE cells, were developed based upon PAINT-derived computational data and were constructed by comparing phylogenetically conserved transcriptional regulation across the four species: human, mouse, rat, and chicken. To establish criteria for selection of specific transcription factors for further analysis, we assigned values to each TRE based upon their frequency of detection across the coordinately expressed gene sets and their evolutionary conservation. An evolutionary conservation factor (ECF) of 1 to 3 points was assigned to each TF, where 1 indicates presence on human genes, 2 indicates human and mouse or rat genes, and 3 indicates a presence on human and chicken as well as either mouse or rat genes. In addition, TREs were scored according to a frequency ratio (FR) derived from the ratio of the percent occurrence of a given TRE in a specific gene subset divided by its frequency of occurrence in the background gene set, for the human genome data. Ultimate TRE selection was based upon criteria filters of a combined ECF score of 2 or greater along with a FR greater than 3.

### Cell culture

Primary cultures of chick embryo RPE cells (cRPE) were established from RPE tissues obtained from fertile White Leghorn chicken eggs maintained in a humidified atmosphere at 37 °C until embryonic day 10, corresponding to Hamburger and Hamilton stage 36 [19,20]. Eyes were enucleated, the anterior segment and vitreous were removed, and the posterior eyecup divided in half and incubated in HBSG (HEPES buffered saline with glucose containing 1 mg/ml glucose, 10 mM HEPES, pH 7.4, 3 mM KCl, and 0.15 mM NaCl). The neural retina was removed and the eyecup was incubated in HBSG containing 20 mM EDTA (ethylene diamine tetraacetic acid) for 1 h. The eye cups were then rinsed for an additional 30 min in HBSG and the RPE was dissected from the choroid. RPE tissue was collected by gentle centrifugation and resuspended in MEM (Eagle's minimum essential medium; Sigma-Aldrich, St. Louis, MO) supplemented with 10% FBS (Fetal Bovine Serum; Sigma-Aldrich), 0.22% sodium bicarbonate and 1% penicillin, streptomycin and amphotericin (Gibco/Invitrogen, Carlsbad, CA). The RPE tissue was mechanically dissociated into single cells and plated onto plastic six well tissue culture plates at a density of 1 eye/well. The plates were previously coated with 1.1 μg/cm^2^ mouse laminin (Sigma-Aldrich) 12 h before cell plating. The cultures were maintained in a humidified atmosphere of 5% CO_2_ at 37 °C for 48 h at which time all non-adherent cells were removed by rinsing with fresh medium [21].

The human RPE-derived cell line ARPE-19 [22] was maintained cultured in DMEM-F12 (Catalog number D8900; Dulbecco's modified eagles minimum nutrient mixture F12 Ham; Sigma-Aldrich) supplemented with 5% FBS, 2 mM L-glutamine, 0.348% sodium bicarbonate and 1% Antibiotic-Antimycotic in T25 culture flasks in a humidified atmosphere of 5% CO_2_. To maintain cells in an undifferentiated state, they were passaged before obtaining confluence. To obtain differentiated cells, cells were grown to confluence and then maintained in DMEM-F12 as above except that the serum was reduced to 1% [23]. These cultures reach confluence 2–3 weeks after passaging and differentiate within 4–6 weeks, though the cultures can be kept in a differentiated state for extended culture periods. After 4–6 weeks in culture the cells exhibit hexagonal packing of pigmented, polarized epithelia and expression of CRALBP and RPE65 typical of morphological and biochemical markers, respectively, of RPE cells in vivo [22].

### RNA extraction and reverse transcriptase polymerase chain reaction amplification

Total RNA was extracted from all cell types using the Micro-to-Midi Total RNA Purification System (Invitrogen, Carlsbad, CA) following manufacturers specifications for isolation of RNA from animal tissues. Purity and concentration of RNA from each sample was assessed on a spectrophotometer at 260 nm wavelength (A_260_). All primers used in this study are listed in Appendix 1 and were designed using the web-based tool GeneFisher Primer Design program, and all ranged between 20 and 22 nucleotides in length, with melting temperatures of 50–65 °C and G-C content between 40%–60% [24]. Primer specificity was determined by the nucleotide-nucleotide basic local alignment search tool (BLASTn) set to the specific species genome, with acceptance of a primer pair based upon expect-value of less than 1 (e-value <1) [25]. RT–PCR was performed using the SuperScript III One-Step RT–PCR System with Platinum Taq (Invitrogen, Carlsbad, CA) according to the manufacturer's instructions. The reaction parameters were (a) cDNA synthesis, 1 cycle at 55 °C for 30 min, (b) denaturation, 1 cycle at 94 °C for 2 min, (c) amplification, 40 cycles at 94 °C for 15 s, (d) melting at primer-specific temperatures for 30 s, (e) extension, 68 °C for 1 min, and (f) final extension, 1 cycle at 68 °C for 5 min. RT–PCR products were analyzed by agarose gel electrophoresis on 1.25% gels containing ethidium bromide (1 μg/ml). The resultant bands were visualized and recorded under ultraviolet light using the Kodak 1D photo system.

## Results

### Transcriptional regulatory network analysis of differentially expressed retinal pigment epithelium genes reveals gene set-specific clusters of transcription response elements

**Figure 1:**
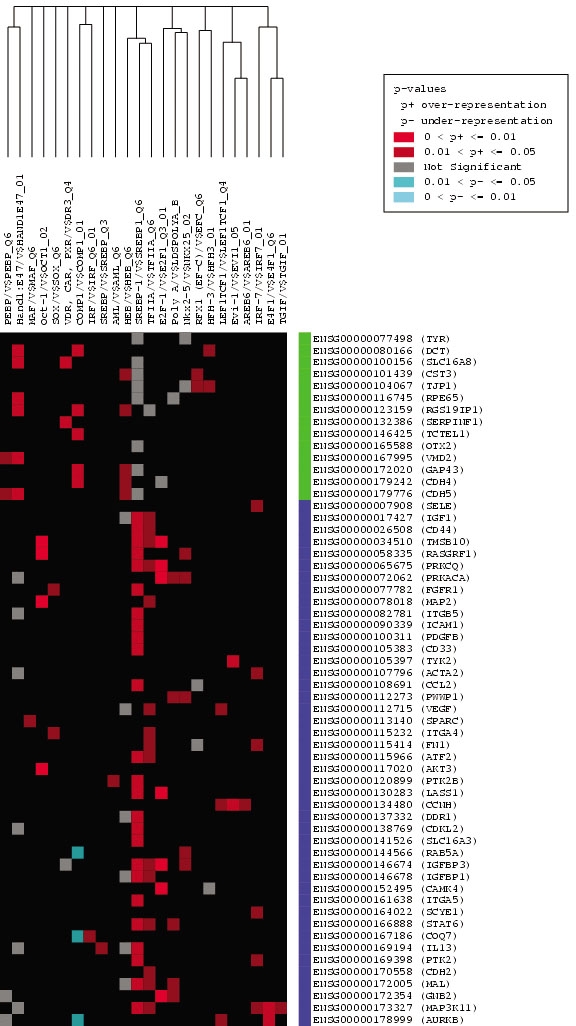
Candidate interaction matrix for statistically enriched transcription response elements from promoter analysis and interaction network toolset analysis of human gene promoters. The retinal pigment epithelium (RPE) gene set was analyzed by promoter analysis and interaction network toolset (PAINT) and a graphic candidate interaction matrix (CIM) was generated as described in Methods. The y-axis lists the Ensembl Gene identifiers for each gene and the x-axis lists the TRANSFAC identifiers for each transcription response element (TRE) found at least once in the promoter region of one or more genes. Genes listed along the y-axis are divided into two clusters that are either upregulated (blue) or down-regulated (green) during epithelial-mesenchymal transformation (EMT) of RPE cells. TREs listed along the x-axis are clustered according to related occurrence pattern calculated using Jaccard's coefficient. The elements within the matrix are color-coded based on the p-value of each TRE found in the regulatory regions of the genes. A red dot represents a TRE that is statistically significant and therefore over-represented in our gene set, while a blue dot signifies an under-represented TRE and a gray dot stands for a TRE with no statistical significance in our gene list. This figure represents the subset of enriched TREs for the human genome; the full CIMs for human and other genomes analyzed are shown in Appendix 4, Appendix 5, Appendix 12, and Appendix13.

**Figure 2:**
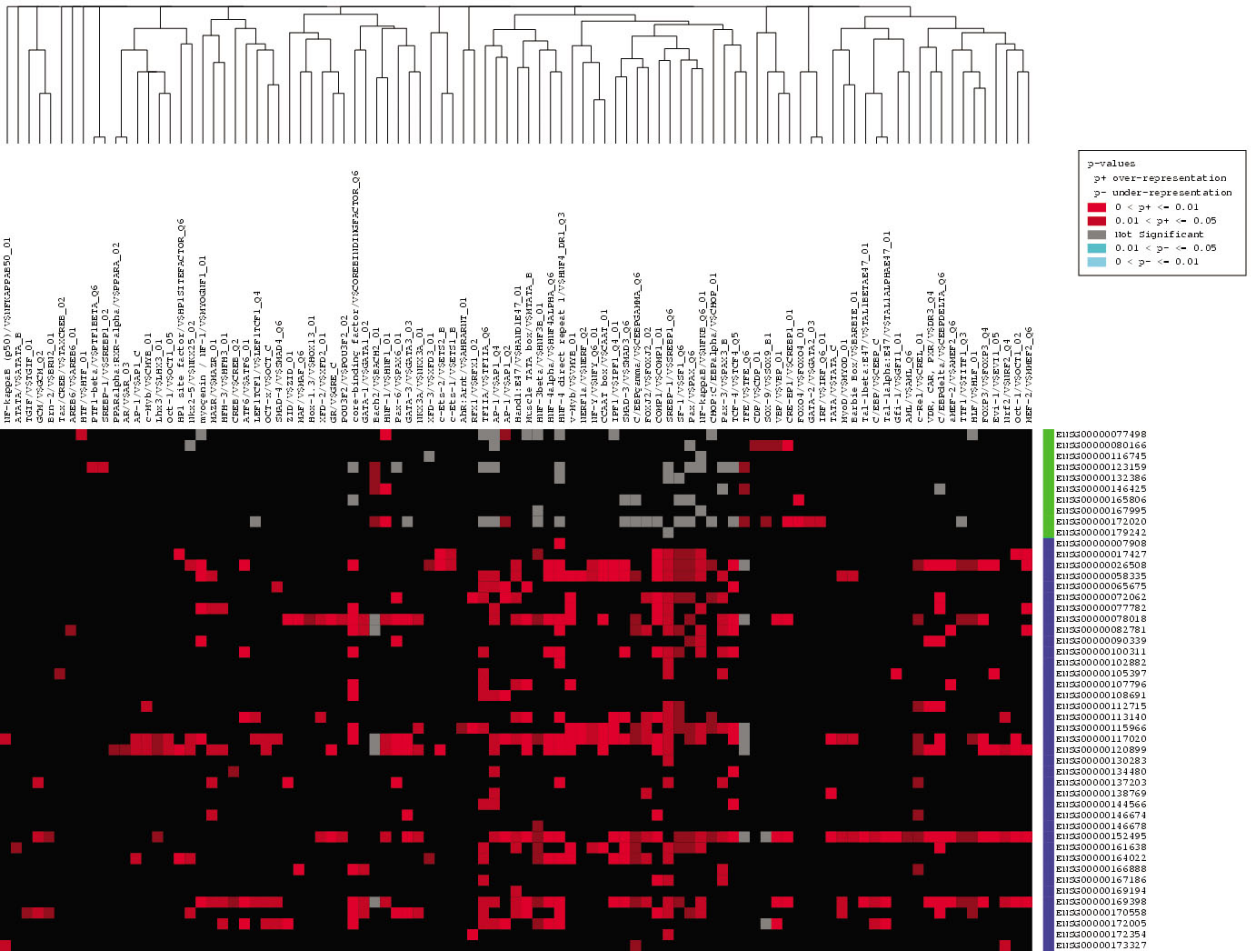
Candidate interaction matrix for statistically enriched transcription response elements from promoter analysis and interaction network toolset analysis of human gene first introns. The retinal pigment epithelium (RPE) gene set was analyzed by promoter analysis and interaction network toolset (PAINT) and a graphic candidate interaction matrix (CIM) was generated as described in Methods. The y-axis lists the Ensembl Gene identifiers for each gene and the x-axis lists the TRANSFAC identifiers for each transcription response element (TRE) found at least once in the first intron region. Genes listed along the y-axis are divided into two clusters that are either upregulated (blue) or down-regulated (green) during EMT of RPE cells. TREs listed along the x-axis are clustered according to related occurrence pattern calculated using Jaccard's coefficient. The elements within the matrix are color-coded based on the p-value of each TRE found in the regulatory regions of the genes. A red dot represents a TRE that is statistically significant and therefore over-represented in our gene set, while a blue dot signifies an under-represented TRE and a gray dot stands for a TRE with no statistical significance in our gene list. This figure represents the subset of enriched TREs for the human genome; the full CIMs for human and other genomes analyzed are shown in Appendix 14 through Appendix 17.

To identify target genes for the analysis of transcription response elements by PAINT, a gene set containing two clusters representing those genes whose expression is preferentially associated with the undifferentiated versus differentiated state of the ARPE-19 cells was established (Table 1). Thus each gene cluster would be predicted to be preferentially associated with one or more TREs involved in their respective coordinate regulation of expression. The genes within the differentiated and undifferentiated clusters were separately analyzed using PAINT to identify and statistically analyze the occurrence of TREs within the promoter region, including 5,000 bp of sequence upstream from the transcription start site, as well as the entire first intron sequence. The complete results of this analysis by the FeasNet Builder module of PAINT are listed in Appendix 2 for the promoter regions and Appendix 3 for the first introns. The analysis identified those TREs that are statistically over-represented on the promoters and first introns of genes within either of the two gene clusters, as compared to their occurrence with the larger background set of promoter and intron regions. TREs with a p-value of less than 0.1 were deemed significant and included for further analysis. These data were then converted into graphic representations of the TRE network or CIM, using the FeasNet Viewer module of PAINT, as illustrated in Figure 1 for the promoter regions and in Figure 2 for the first introns. Inspection of the matrix patterns for the respective differentiated and undifferentiated gene clusters indicated that distinct subsets of TREs were associated with each of these two clusters, suggesting that these TREs could represent components of the networks regulating coordinated expression of genes within each cluster. Figure 1 and Figure 2 represent a subset of the complete CIM, and include only those TREs with a p-value of less than 0.1. The complete CIMs representing the results of the analysis of the full 60 gene set are tabulated in Appendix 2 and Appendix 3, as well as illustrated in Appendix 4 and Appendix 5. The results of the CIM analysis indicate that several specific TREs are statistically overrepresented within the regulatory regions of genes within each gene subset, and furthermore that several of these TREs differ between the two subsets. These distinctly represented TREs are thus candidates for further analysis regarding their potential regulatory role in coordinating gene expression during RPE cell differentiation.

**Figure 3:**
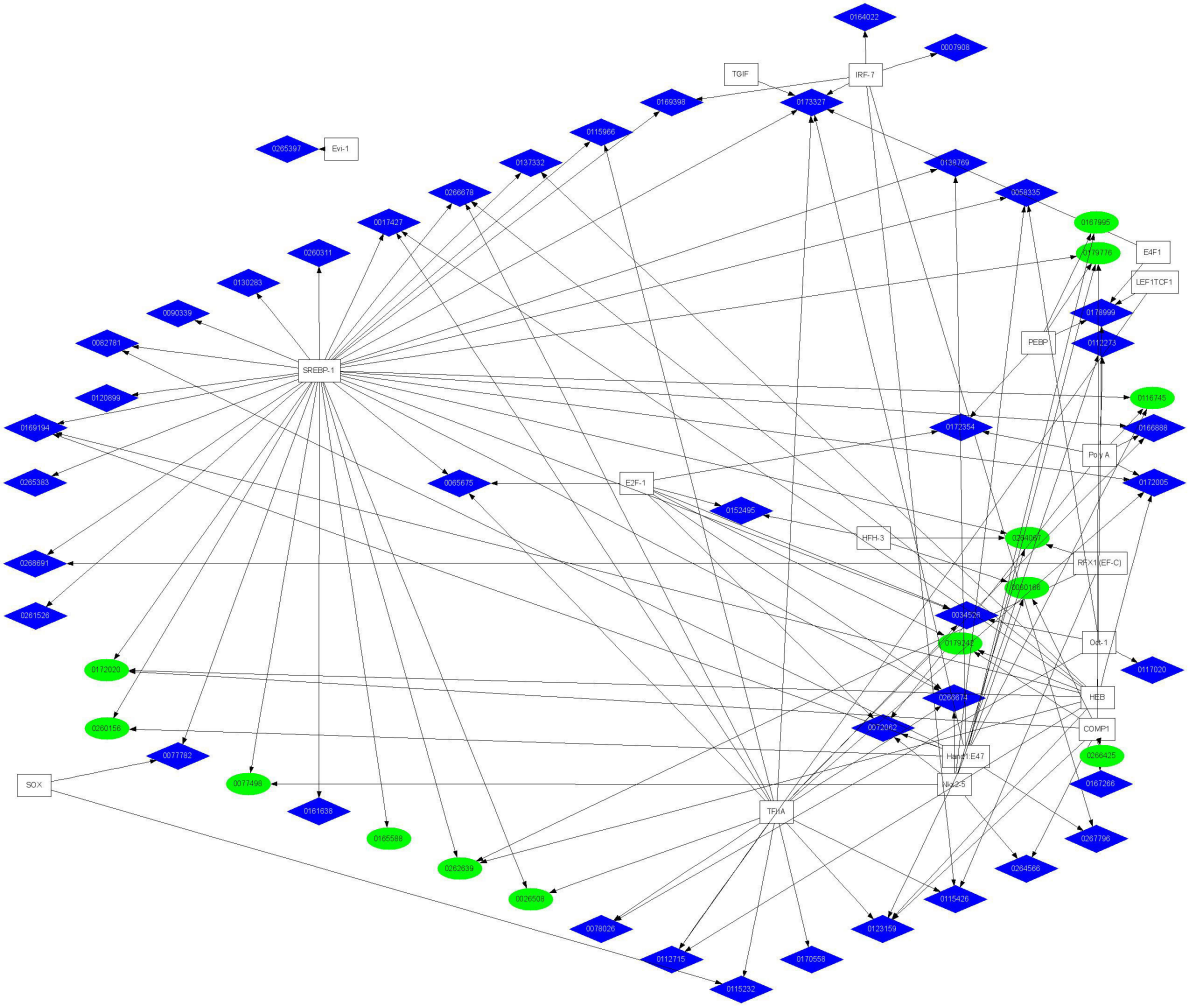
Transcriptional regulatory network diagram for transcription response element associated with human promoter regions. The graphical representation was derived using GraphViz as described in Methods. The ellipses and diamonds represent individual genes divided into upregulated (blue) and down-regulated (green) clusters. The boxes represent TREs, with arrows indicating gene-TRE associations. Corresponding network diagrams for mouse, rat and chick promoter regions are shown in Appendix 18 through Appendix 20.

**Figure 4:**
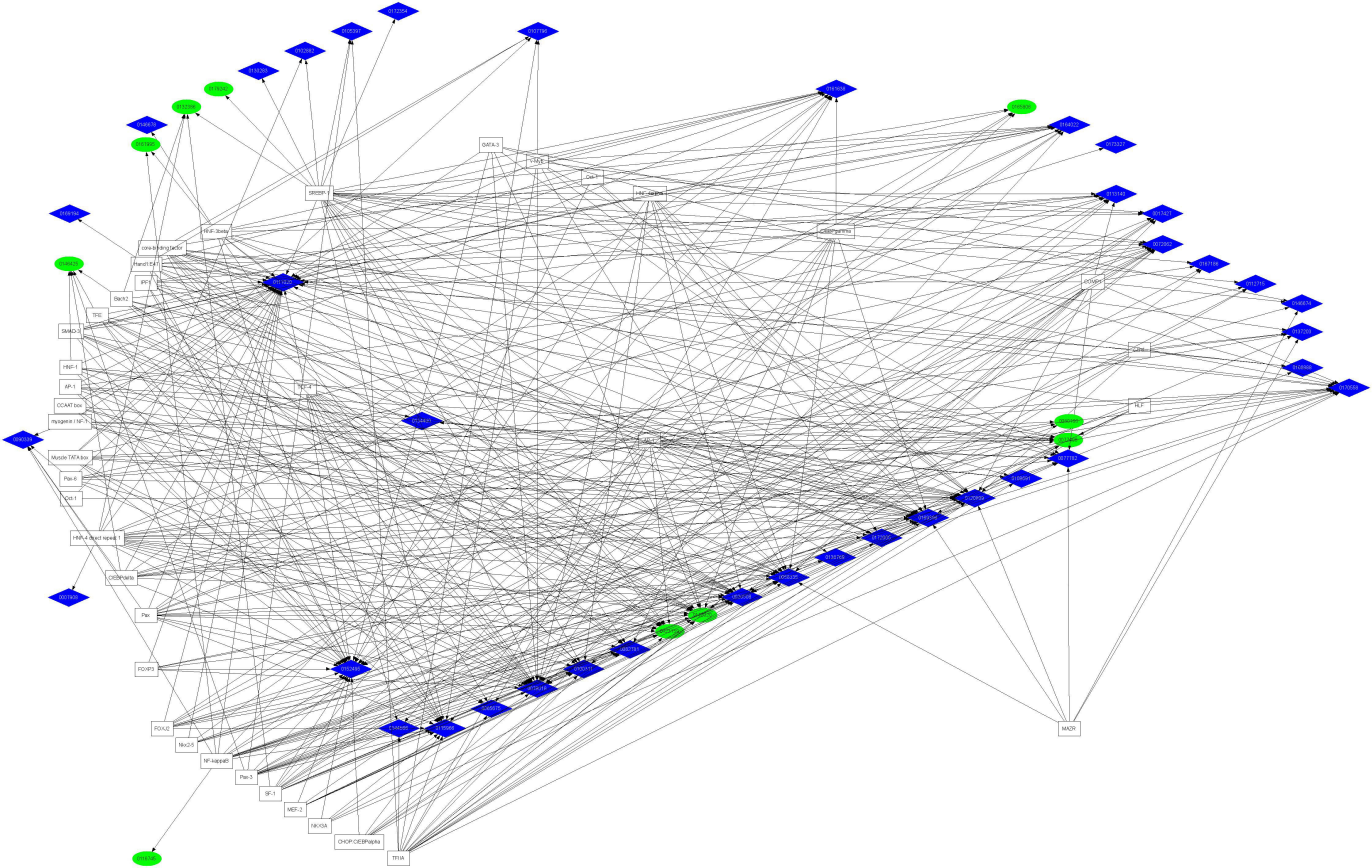
Transcriptional regulatory network diagram for transcription response element associated with human first intron regions. The graphical representation was derived using GraphViz as described in Methods. The ellipses and diamonds represent individual genes divided into upregulated (blue) and down-regulated (green) clusters. The boxes represent TREs, with arrows indicating gene-TRE associations. Corresponding network diagrams for mouse, rat and chick first intron regions are shown in Appendix 21 through Appendix 23.

To further visualize the potential interrelationships of the candidate TREs and genes within each cluster, the data generated by the FeasNet Viewer module was graphically presented using GraphViz to generate a regulatory network diagram for the human gene set, as illustrated in Figure 3 for the promoter regions and Figure 4 for the first introns. These results identify nodes within each network and further identify those TREs with the potential to coordinately regulate subsets of genes within each cluster. The TREs that comprise the nodes of this visualization represent all those TREs which are overrepresented among the genes of each cluster, and were found to be associated with as few as one gene within each cluster to as many as 28 genes within a cluster. Those TREs exhibiting high levels of interconnectivity are among the best candidates for coordinate regulation of RPE genes.

**Figure 5:**
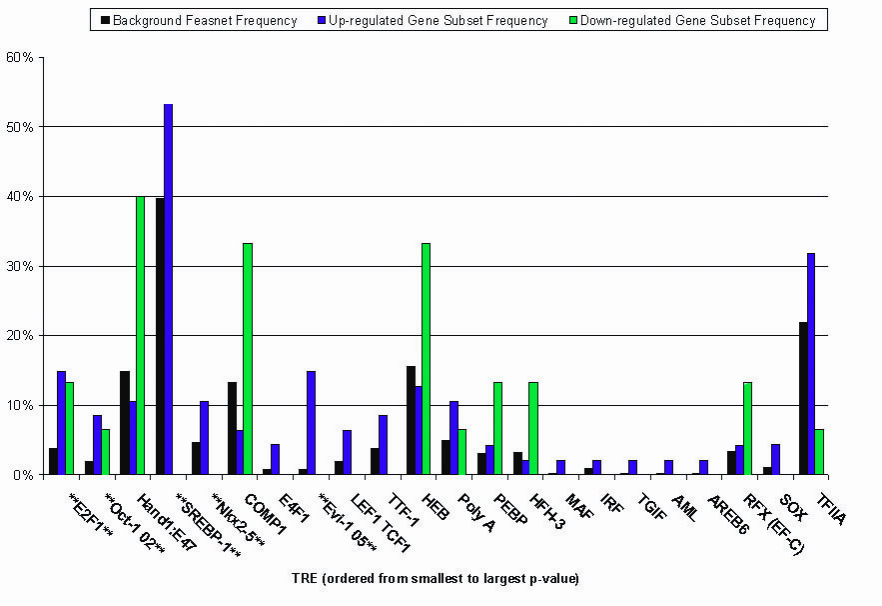
Frequency analysis of transcription response element representation in human promoter regions. Frequency of occurrence of each transcription response element (TRE) in the human gene promoter regions was determined from the promoter analysis and interaction network toolset (PAINT) analysis as described in Methods. The y-axis indicates the frequency of each TRE among the upregulated (blue) or down-regulated (green) gene clusters as well as among the full background gene set (black). The x-axis indicates the over-represented TREs, ordered by increasing p-value. Corresponding frequency analyses for mouse, rat and chick promoter regions are shown in Appendix 24 through Appendix 26.

**Figure 6:**
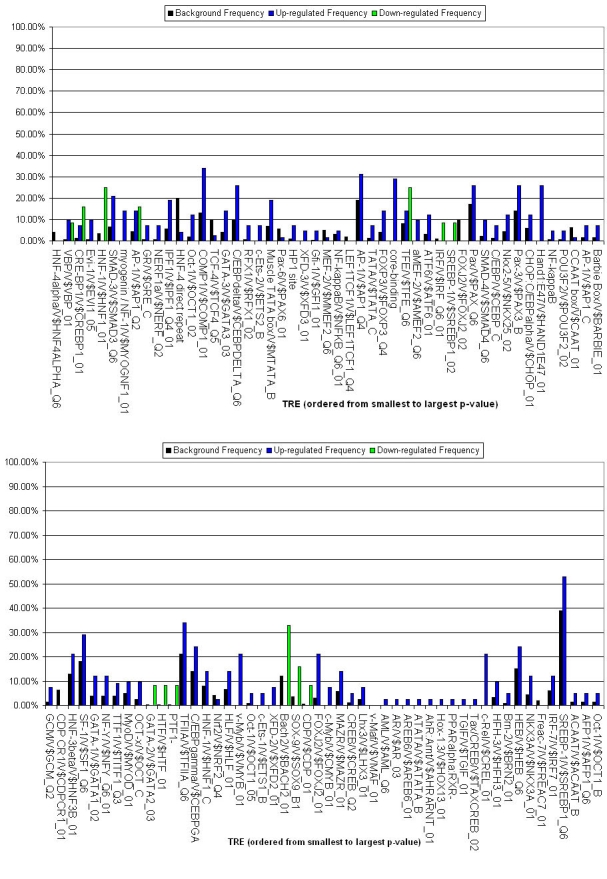
Frequency analysis of transcription response element representation in human first intron regions. Frequency of occurrence of each transcription response element (TRE) in the human gene first intron regions was determined from the PAINT analysis as described in Methods. The y-axis indicates the frequency of each TRE among the upregulated (blue) or down-regulated (green) gene clusters as well as among the full background gene set (black). The x-axis indicates the over-represented TREs, ordered by increasing p-value. Corresponding frequency analyses for mouse, rat and chick first intron regions are shown in Appendix 27 through Appendix 29.

To identify those TREs of particular significance in the differential regulation of RPE genes during EMT, we next performed an analysis comparing all the overrepresented TREs from the CIM analysis with respect to their relative frequency of occurrence between the differentiated, undifferentiated and background gene clusters. While the previous analyses identified TREs overrepresented in either or both of the two gene clusters, the frequency analysis further distinguished those TREs preferentially associated with either the differentiated or undifferentiated gene cluster. The results, which are shown in Figure 5 for the promoter regions and Figure 6 for the intron regions, indicate that a select subgroup of the TREs can be assigned as potential regulators of one of each of the two cell states. For example, in the promoter regions, Hand1:E47 and COMP1 show increased relative frequency among down-regulated genes, whereas Oct-1 and SREBP-1 do so among upregulated genes. In the first intron regions, CDP and IRF show increased relative frequency among down-regulated genes, whereas GATA-3 and MAZR do so among upregulated genes.

### Phylogenetic comparison of PAINT analyses identifies evolutionarily conserved transcriptional regulatory elements across divergent species

**Figure 7:**
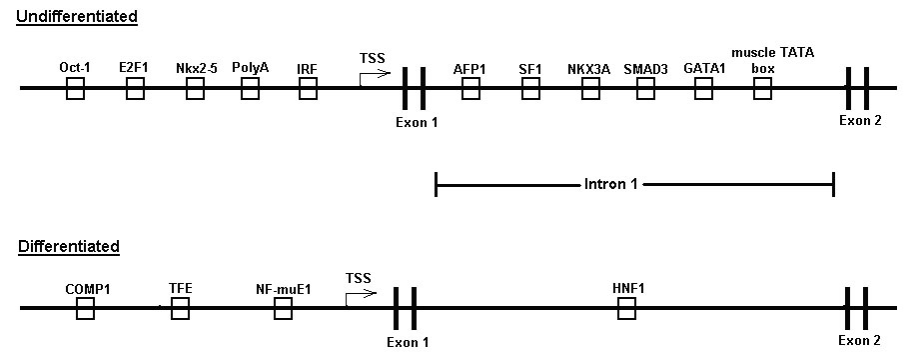
Archetypal cross-species gene regulatory region models of undifferentiated and differentiated gene clusters. These models incorporate transcription response element (TREs) that were found to be over-represented in results of both the human and chicken, as well as either the mouse or rat, promoter analysis and interaction network toolset (PAINT) analysis. TSS represents transcriptional start site.

The above studies of gene regulatory networks regulating RPE cell differentiation were focused on human genome sequence analysis. However, evolutionary conservation analysis of regulatory regions can also be useful for identifying functionally important sites. Therefore, to further identify those elements of these hypothetical networks that would most likely be of functional significance due to their evolutionary conservation, we performed parallel PAINT analyses similar to those described above for human genomic elements for three additional model species: mouse (*Mus musculus*), rat (*Rattus norvegicus*), and chicken (*Gallus gallus*). The results of the FeasNet Builder analysis in the three additional species gene sets indicate that, as observed for the analysis of human genes, several overrepresented TREs are again identified (Appendix 6, Appendix 8, and Appendix 10 for the promoter regions and in Appendix 7, Appendix 9, and Appendix 11 for the first introns) for each species. The graphical candidate interaction matrix for each of these three species is shown in Appendix 12, Appendix 13, Appendix 14, Appendix 15, Appendix 16, and Appendix 17, while the GraphViz output of the FeasNet Viewer analyses illustrating the regulatory network diagrams for these three species is shown in Appendix 18, Appendix 19, Appendix 20, Appendix 21, Appendix 22, and Appendix 23, which both again indicate the presence of a complex regulatory network within each of these species. As for the human genome, we also compared the frequency of occurrence of each identified TRE between each gene cluster and the background gene set for each of the three model species. These frequency analyses are shown in Appendix 24, Appendix 25, Appendix 26, Appendix 27, Appendix 28, and Appendix 29. As for the human genome analysis discussed above, these results of the CIM, GraphViz and frequency analyses identify TREs for each of the three species which are also candidates for regulation of gene expression during RPE differentiation. Comparison of the results from the human as well as model system analyses identified both phylogenetically conserved as well as species-specific TREs, which were incorporated into criteria tables and gene regulatory region models as described below (Table 2 and Figure 7).

**Table 2:** Criteria table for transcription response element inclusion in gene regulatory network models

**TRE**	**p-value**	**Evolutionary conservation factor**	**Frequency ratio**
Promoter			
E2F-1/V$E2F1_Q3_01	0.00095	2	3.725
Oct-1/V$OCT1_02	0.00801	3	4.25
Nkx2–5/V$NKX25_02	0.05404	2	2.3
Poly A/V$LDSPOLYA_B	0.07291	2	2.16
IRF/V$IRF_Q6_01	0.08969	2	2.33
COMP1/V$COMP1_01	0.03569	2	2.52

First intron			

Evi-1/V$EVI1_05	0.00003	2	13.86
Oct-1/V$OCT1_02	0.00043	2	6.32
GR/V$GRE_C	0.00045	2	14.6
SMAD-3/V$SMAD3_Q6	0.00071	3	3.18
HNF-1/V$HNF1_01	0.00918	2	7.35
AP-1/V$AP1_C	0.01893	2	4.87

Represented for each transcription response elements (TRE) identified in the promoter and intron regions of each gene are the TRANSFAC identifier name, the p-value, the evolutionary conservation factor (ECF), and the frequency ratio (FR) as defined in Methods. TREs included in this table are those with p-value <0.1, ECF=2, and FR>3. The full table of values for all TREs analyzed is contained in Appendix 30 (promoters) and Appendix 31 (introns).

### Compilation of human and cross-species transcriptional regulatory network analyses generates global as well as gene-specific regulatory models

To generate a global working model of gene expression regulation in the RPE, we compared the above results across the four species analyzed and identified those TREs of highest evolutionary conservation, as well as highest frequency of occurrence, by calculation of an evolutionary conservation factor (ECF) score, as well as a frequency ratio (FR) score, for each TRE identified in the computational analysis, as described in Methods. The results of these calculations are shown in Table 2, with the table containing only those results for TREs that passed the three criteria of a p-value >0.1, an ECF greater or equal to 2, and an FR greater or equal to 3. The full data set of calculated ECFs and FRs for all the TREs identified in the computational analysis is shown in Appendix 30, for the promoter regions and Appendix 31 for the first intron regions. These calculations provide a scheme for ranking TREs, and by extension their corresponding TFs, for inclusion into models of RPE gene regulation, as well as for further studies probing their expression and function in RPE differentiation.

**Figure 8:**
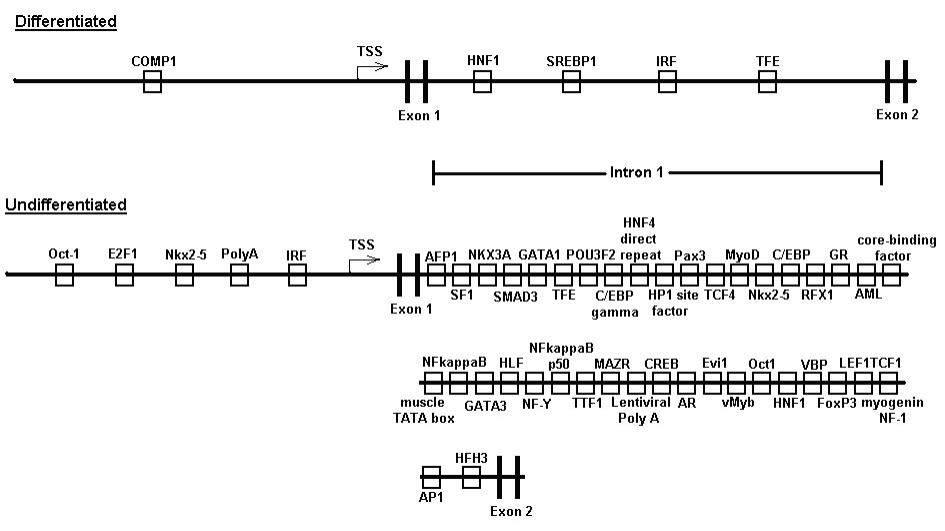
Human gene regulatory region models of undifferentiated and differentiated gene clusters. These models incorporate transcription response elements (TREs) that were found to be over-represented in results of the human, and either the chicken, mouse or rat, promoter analysis and interaction network toolset (PAINT) analysis. TSS represents transcriptional start site.

**Figure 9:**
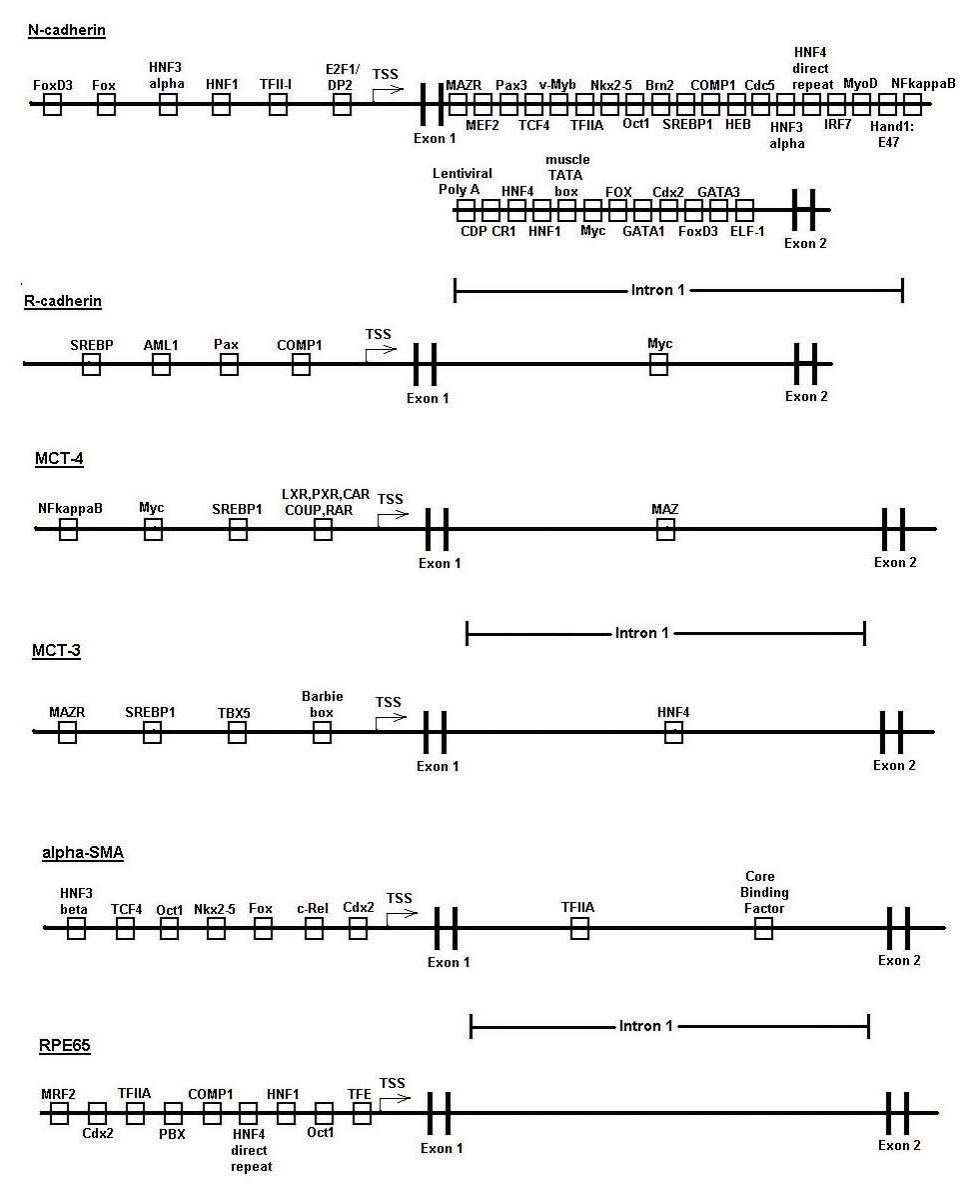
Gene regulatory region models for specific reciprocally-regulated gene pairs. Models for the paired genes that are reciprocally regulated during EMT of RPE cells models including N- and R-cadherin (**A**), α-SMA and RPE-65 (**B**), and MCT-3 and −4 (**C**). Models were constructed by including only those TREs that are over-represented in both the human and chicken, as well as either the mouse or rat, PAINT analysis.

The TREs passing all these criteria, and hence representing potentially evolutionarily conserved regulators of RPE gene expression, were incorporated into archetypal gene models for the coordinated up- and down-regulation of genes during RPE cell differentiation, as shown in Figure 7. Although evolutionary conservation is a potential indicator of conserved function, in recognition of the likelihood that species-specific aspects to coordinated gene regulation also exist, we also developed an additional model with modified ECF criteria focused on regulation in the human genome, such that incorporation of TREs into this second model was dependent on the TRE occurring within the human gene set and any one additional species, while maintaining the same p-value and FR criteria filters. The resulting models are shown in Figure 8, and share many elements of the trans-species model, in some cases eliminating TREs not found in the human analysis, while adding some others due to the less stringent ECF criteria. Finally, additional gene-specific regulation models were constructed for selected genes representing paired members of multigene families, or other markers, with reciprocal expression during RPE cell differentiation. These paired models were constructed for the cell adhesion proteins N- and R-cadherin, for the lactate transporters MCT3 and −4, and for α-smooth muscle actin and RPE65 (Figure 9). These gene regulation models identify those TREs most likely to coordinate expression of specific genes as well as broader sets of up- and down-regulated genes during RPE cell differentiation, and provided targets for validation of these models through direct biochemical analysis as described below [22,23,26].

### Validation of gene models by identification of transcription factors whose expression is dependent upon the state of retinal pigment epithelium cell differentiation

**Figure 10:**
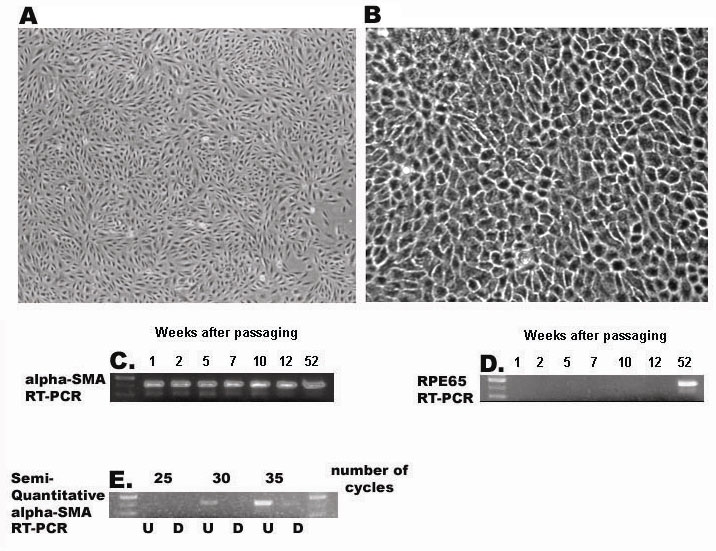
Reverse transcriptase polymerase chain reaction analysis of markers during retinal pigment epithelium cell differentiation. mRNA was isolated from undifferentiated (**A**) or differentiated (**B**) ARPE-19 cells and subjected to reverse transcriptase polymerase chain reaction amplification to detect SMA (**C**) or RPE65 (**D**) as described in Methods. Phase-contrast micrographs represent undifferentiated (**A**) or differentiated (**B**) ARPE-19 cells after one week (**A**) or 52 weeks (**B**) of culture. **C** and **D** represent RT–PCR amplification of mRNA samples isolated from ARPE-19 cells maintained in culture for 1, 2, 5, 7, 10, 12, and 52 weeks, using primers to detect mRNA for either αSMA (**C**) or RPE65 (**D**). **E** represents RT–PCR amplification for a series of 25, 30, or 35 cycles to detect αSMA using mRNA isolated from ARPE-19 cells that are undifferentiated (U) differentiated (**D**). The first lane in **C-E** represents a DNA standard ladder of 300, 400, and 500 bp.

**Figure 11:**
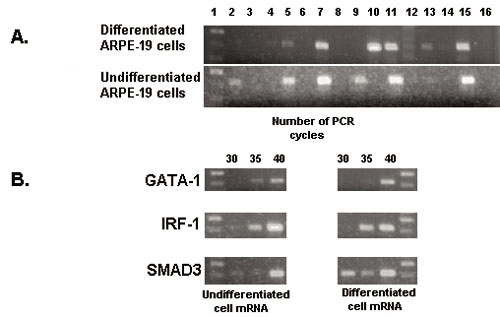
Reverse transcriptase polymerase chain reaction amplification of transcription response element mRNAs during ARPE-19 retinal pigment epithelium cell differentiation RNA was isolated from undifferentiated and differentiated ARPE-19 cells and subjected to RT–PCR analysis to detected transcription response element (TRE) mRNAs as described in Methods. In **A**, all reactions were performed for 40 cycles, where lane 1 represents DNA standards, lane 15 represents the positive control primers for GADPH, and lane 16 is the negative control with no mRNA template. The intervening lanes in **A** represent primers specific for the following TFs: 2 Core binding factor, 3 E2F1, 4 Evi-1, 5 GATA1, 6 HNF-1, 7 IRF-1, 8 Nkx2–5, 9 NKX3A, 10 Oct-1, 11 SMAD3, 12 SREBP-1, 13 TFE3, 14 v-Myb. In **B**, semi-quantitative RT–PCR was also done for either 30, 35, or 40 cycles as indicated using primers specific for GATA-1, IRF-1, or SMAD3. The first lane in **A** represents a standard DNA ladder at 300, 400, and 500 bp, while in **B** the DNA standards are at 400 and 500 bp.

**Figure 12:**
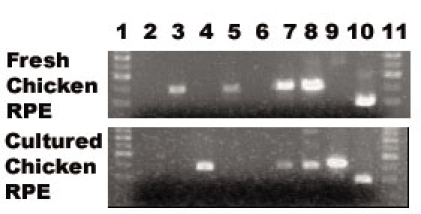
Reverse transcriptase polymerase chain reaction amplification of transcription response elements mRNAs during chick embryo retinal pigment epithelium cell differentiation RNA was isolated from undifferentiated cultured chick embryo retinal pigment epithelium (RPE) cells or differentiated fresh RPE tissue and subjected to RT–PCR analysis to detect transcription response elements (TREs) mRNAs as described in Methods. Lanes 1 and 11 represent DNA standard ladders at 300, 400, 500, 600, and 700 bp, lane 10 represents the positive control for GADPH, and the remaining lanes represent primers specific for the following TFs: 2 FoxD3, 3 AML-1, 4 HNF-3α, 5 HNF-1, 6 E2F1, 7 DP1, 8 TFII-I, 9 SREBP-1.

The above gene regulatory models identified TREs that could serve as elements of the gene regulatory network during RPE cell differentiation. For these TREs to play a role in regulation of their associated genes, the activity of the corresponding transcription factors would be expected to be dynamically regulated at appropriate times to effect such control. One common mechanism of regulation for TF activity is at the transcriptional level resulting in differential steady-state mRNA expression levels. Thus, to test the computationally derived models, we used RT–PCR to assay for the presence of mRNA encoding transcription factors (TFs) predicted to play a regulatory role in the RPE. The results obtained from comparison of mRNA extracted from undifferentiated and differentiated RPE cells, using both the human ARPE-19 cell line and primary embryonic chicken RPE cells, are shown in Figure 10, Figure 11, Figure 12. The analyses of ARPE-19 cells were performed using both undifferentiated cells that been in culture for one month, exhibiting morphology of fusiform, unpigmented mesenchymal cells, as well as differentiated ARPE-19 cells exhibiting morphology of polygonal, darkly pigmented, epithelial cells (Figure 10A,B). RT–PCR was first performed on each respective cell population targeting mRNAs for α-SMA and RPE65, respective markers of the mesenchymal undifferentiated and epithelial differentiated state of RPE cells [22]. These results indeed demonstrated that mRNA encoding α-SMA, but not RPE65, was expressed in undifferentiated cells, whereas RPE65 mRNA was readily detected among differentiated cells with a reduced level of α-SMA mRNA (Figure 10C,D). Semi-quantitative RT–PCR was used to further distinguish these levels of α-SMA mRNA, which more clearly distinguished these two cell states (Figure 10E). Having verified that these two markers were distinctly expressed between these two cell populations, we then used RT–PCR to determine the levels of mRNA encoding the specific transcription factors previously identified by the computational analysis, which were expected to fall into three categories, exhibiting either quantitative, qualitative, or no differences between the two test cell populations. Of these TFs, mRNAs encoding four were found to be reciprocally expressed in differentiated versus undifferentiated ARPE-19 cells, with Oct-1 and TFE3 detected only in differentiated cells, and Core Binding Factor and NKX3A detected only in undifferentiated cells (Figure 11). mRNAs encoding additional transcription factors, including GATA-1, IRF-1, and SMAD3, were detected in both cell states (Figure 11A). Semi-quantitative RT–PCR was then used to further analyze differences in the expression patterns of these factors, with GATA-1 detected at higher levels in differentiated cells, whereas IRF-1 and SMAD3 were detected at higher levels in undifferentiated cells (Figure 11B).

**Figure 13:**
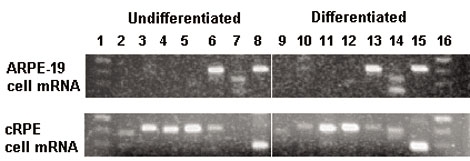
Reverse transcriptase polymerase chain reaction amplification of EMT-associated transcription response elements mRNAs during ARPE-19 and chick embryo retinal pigment epithelium cell differentiation RNA was isolated from undifferentiated and differentiated ARPE-19 or chick embryo retinal pigment epithelium (cRPE) cells and subjected to RT–PCR analysis to detect transcription response elements (TRE) mRNAs as described in Methods. Lanes 1 and 16 represent DNA standard ladders at 300, 400, and 500 bp, lanes 8 and 15 represent the positive controls for GADPH, and the remaining lanes represent primers specific for the following TFs: 2 and 9, Slug; 3 and 10, Snail; 4 and 11, Twist; 5 and 12, SIP-1; 6 and 13, SMAD-2; 7 and 14, LEF-1.

Similar analyses were performed using freshly isolated chicken RPE tissues and primary chick RPE cultures. When cultured, chick RPE cells re-enter the cell cycle and de-differentiate [27]. RNA was prepared from freshly isolated RPE cells as well as from cells cultured for five days in vitro, and both cell populations were probed for TFs corresponding to those TREs identified in association with both the global as well as gene-specific regulatory models. The results indicated that, similar to ARPE19 cells, and consistent with model predictions, the primary chick RPE cells also revealed reciprocal TF expression between differentiated and undifferentiated cells. mRNAs encoding AML-1 and HNF-1 were detected only in the differentiated chicken RPE cells, whereas mRNAs encoding HNF-3 and SREBP-1 were detected only in the undifferentiated cells, while two additional mRNAs encoding TFs DP-1 and TFII-I were detected in both cell populations (Figure 12). Finally, as an adjunct to the PAINT-derived analyses, an additional series of RT–PCR reactions were performed to determine whether other TFs, not identified through PAINT, but known from prior studies to be involved in EMT of epithelial cells other than RPE, were expressed in chick or ARPE-19 cells. As shown in Figure 13, RT–PCR amplification of mRNA from undifferentiated and differentiated primary chick RPE cells generated similar levels of amplicons for Slug, Twist and SIP1, whereas Snail was detected at higher levels in undifferentiated cells, and LEF1 was detected only in differentiated cells. When similar analyses were performed with total RNA isolated from differentiated or undifferentiated ARPE-19 cells, Slug, Snail, Twist, and SIP1 were not detected, while SMAD2 was detected at equal levels in both samples. Interestingly, while LEF-1 was also detected in both cell populations, a distinct additional amplicon was detected in differentiated RPE cells, indicating that differential splicing of this gene transcript occurs during the course of RPE differentiation. Our inability to detect expression of certain classical mediators of EMT such as Snail, Slug, Twist or SIP1 in ARPE-19 cells, while we were able to detect them in primary cultures of embryonic chick RPE cells, may be related to the different stages of development represented by these two model systems (embryonic versus adult), to the unique properties of RPE cells compared to other epithelial cell types that may exhibit species-specific differences, or to some specific phenotypic property of ARPE-19 cells that arose during their derivation [28,29]. Overall, the results of the RT–PCR analyses indicate that the computational biology approach was successful at identifying transcription factors whose expression is regulated during RPE cell differentiation, and thus may play a role in control of differential gene expression and modulation of RPE cell phenotype.

## Discussion

**Figure 14:**
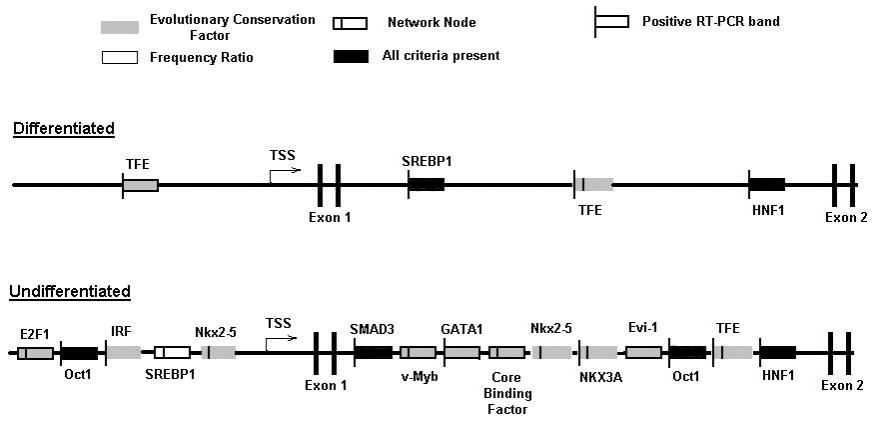
Comprehensive cross-species models for reciprocal regulation of genes during retinal pigment epithelium cell differentiation Models for regulatory regions, including promoters and first introns, of differentiated and undifferentiated gene clusters, were constructed as described in the text. transcription response elements (TREs) inclusion criteria indicated in these models are frequency ratio, evolutionary conservation factor, and RT–PCR detection of mRNA expression. Symbols representing TREs indicates passage of corresponding criteria filter, as indicated in key, by TRE, filled boxes signify that a TRE has passed all criteria filters.

The results of the present study have permitted the construction of several hypothetical models for regulation of genes in RPE cells during EMT, each generated using a different set of theoretical boundaries and statistical criteria. While the computational approach using the PAINT toolset has been previously applied to other cell types [12,30], to the best of our knowledge the present work represents the first application to the analysis of RPE cell differentiation. A strength of the models developed here is that they make strong predictions of which TFs would be expected to be differentially acting during phenotypic changes in RPE cells, predictions which were successfully tested and positively borne out by the RT–PCR analyses in the present studies. These results form the basis for design of future studies that will be directed at testing the function of these various TFs in regulating RPE cell phenotype. These experiments are guided by the integration of the experimental results into a comprehensive model for RPE gene regulation (Figure 14), which indicates for each TRE included in the final model, the various criteria filters that led to its inclusion, including evolutionary conservation, frequency of occurrence, position in a gene regulatory network node, and generation of a positive amplicon in RT–PCR validation assays. Two TFs, Oct1 and HNF1, although not previously identified with respect to EMT, pass all of these four criteria, may play a unique role in this context in RPE cells, and thus are identified as excellent candidates for direct functional analysis in future studies.

While not previously analyzed in the context of RPE cells, the transcription factors identified in the present study by the PAINT and RT–PCR analyses can be categorized with respect to other cell types into three groups that include (1) TFs not previously associated with EMT; (2) TFs that, while not previously known to directly affect EMT, have been shown to regulate cellular processes that are components of EMT, and (3) TFs previously shown to directly affect EMT principally in other cell types. The first group includes the TF GATA-1, which of all the TFs identified in this study is the only one not directly linked to an EMT-related process. This factor is expressed in cells of the erythroid lineage and is essential for proper erythroid development, but its potential role in regulation of epithelial cell phenotype remains to be determined [30-32]. The second group encompasses the factors such as Oct-1, HNF-1, NKX3A, IRF-1, SREBP-1, and Core Binding Factor, which have not been specifically linked to EMT, yet regulate processes such as cell migration, cell adhesion and metabolic pathways associated with EMT. Oct-1 and HNF-1 act as important regulators of development processes such as neural tube development [33-36]. NKX3A, a homolog of NKX2–5 that functions to activate N-cadherin expression in cardiac development, may function in a similar manner by activating N-cadherin expression, which has been shown to be highly expressed in metastatic cancer cells [37-41] and is upregulated during RPE de-differentiation. Interferon regulatory factor-1 (IRF-1) is another factor that falls into this category, in that it plays an important tumor suppressive role in a wide variety of human neoplasias [42,43]. Sterol regulatory element binding protein-1 (SREBP-1) is known to affect expression of lipogenic genes in the liver, which is of interest insofar as cells undergoing EMTs possess altered fatty acid and glucose/insulin metabolism [44]. Previous work has shown a switch to aerobic glycolysis when cells begin to migrate in the initial stages of EMT, an action that may be mediated by SREBP-1 [45]. Core binding factor (CBF) may also be indirectly involved in EMT, in that it interacts with members of the TGF-β signaling factor to influence cell growth and differentiation [46]. Finally, SMAD3 and TFE3 constitute the last group and have previously been strongly implicated in the signaling pathways associated with TGF-β induced EMT, whereby they activate LEF-1 transcription, a major EMT inducer [47-51]. These two TFs are thus also identified as excellent candidates for further analysis in RPE cells, since they were identified through the PAINT analysis and have also been previously identified as regulators of genes associated with EMT in several cell types [52-56], and in one report in RPE cells [57]. Thus, while this study has identified several novel potential regulators of the RPE, the concordance between certain results of the present study and prior reports provides further validation of a combined in silico computational approach as an adjunct to in vivo as well as in vitro biochemical and cell biologic studies.

One potential limitation of the present approach is indicated by the apparent lack of identification by the PAINT analysis of some TFs that have been previously associated with EMT in other cell types and that may play a role in RPE as well. These include additional downstream mediators of TGF-β signaling pathways besides SMAD3 such as Snail, Slug, Twist, SMAD2, SIP1, β-catenin, and LEF-1 [55,57-61]. As one approach to addressing this, we performed RT–PCR assays to determine the presence of mRNAs corresponding to these TFs, and did detect several of these in RPE cells, although only LEF-1 was indicated to be differentially expressed between differentiated and undifferentiated cells. Interestingly, differential splicing of LEF-1 mRNA, as detected here, has been reported previously to generate several isoforms that may confer distinct functionality on this TF [62]. The primers used in our RT–PCR flank the third through sixth exons, the third of which encodes a premature stop codon that can generate a form of LEF-1 retaining its β-catenin binding site but lacking DNA binding domain and nuclear localization signal [62]. Given the key role of LEF-1 in the TGFβ signaling pathway, this may indicate one possible means through which modulation of such pathways occurs during RPE differentiation. For any TRE to be analyzed via PAINT, its sequences must be available in TRANSFAC database, and our manual inspection of this database revealed that no sequences are available for TREs corresponding to Slug in any species, and Snail, Twist and SIP1 sequences are available only for the mouse genome, whereas only SMAD-2 and LEF-1 sequences are available for all species analyzed in this study. Of these TREs for which at least partial sequence data was available, although some were indeed detected by PAINT in some genes within the clusters, only LEF-1 was enriched with a p-value <0.1, but had a low ECF value.

A second limitation of the present study is the inherent variability observed in the phenotype of ARPE-19 cells. Several reports have indicated that there is a degree of variability, depending on the culture conditions such as serum concentration and growth substrate, as well as differences between ARPE-19 cells and native human RPE [63,64]. While we acknowledge that this inherent variability exists, the ARPE-19 cells used in the present study were from undifferentiated and well differentiated cell cultures, respectively, as defined by both morphological as well as biochemical criteria.

In situ, the RPE is a monolayer of morphologically and functionally polarized non-proliferative and non-migratory cells whose unique properties are essential to the proper development and function of the retina. However, these cells are known to exhibit a high degree of plasticity in phenotype and function both in vitro and in vivo [1,3]. Delineating the mechanisms underlying this plasticity is essential to understanding the conditions under which RPE cells undergo these changes, and is critical to developing preventive and therapeutic interventions for conditions in which RPE plasticity may lead to retinal diseases such as proliferative vitreoretinopathy (PVR) [6]. Current therapeutic techniques used to treat retinal detachments and their complications are limited to invasive surgical procedures aimed at physically re-attaching the sensory portion of the retina to the underlying RPE, and removing epiretinal membranes, such as laser- or cryo-therapy, supplemented by pneumatic retinopexy, scleral buckling or vitrectomy. Presently, PVR occurs as a complication in up to 10% of surgical retinal detachment repairs, making it the most common post-surgical complication associated with these procedures [4]. Development of non-surgical or adjunct treatments for PVR will require a better understanding of the underlying biology of the genetic and epigenetic mechanisms regulating RPE cell phenotype and underlying the plasticity exhibited by RPE cells. Since this plasticity likely reflects changes in the expression of a wide variety of gene products, and thus ultimately the coordinated function of several transcription factors, the present study was designed to apply the tools of computational biology to identify transcription factors whose function could modulate changes in RPE cell phenotype. The TFs identified in this study thus become excellent candidates for further analysis of their role in this process.

In conclusion, we have predicted and experimentally verified the differential expression of several transcription factors including Oct-1, HNF-1, SMAD3, TFE, Core binding factor, GATA-1, IRF, NKX3A, SREBP-1, and LEF-1 that may be of importance in the regulation of genes during EMT of RPE cells, as determined first by computational analysis and modeling, and then tested by direct RT–PCR analysis. The results clearly indicate that several of these TFs are differentially regulated during RPE differentiation and thus may play a role in epithelial-mesenchymal transformations of RPE cells in both developmental and disease processes. These TFs are thus excellent targets for further studies directed at testing their role as regulators of RPE cell phenotype, and consequently may also provide future targets for therapeutic interventions in cases of PVR and other related disorders of the eye.

## Appendix 1. Primer sequences used for ARPE-19 and Chicken Primary RPE cells RT-PCR amplification of selected transcription factor mRNAs.

To access the data, click or select the words “Appendix 1.” This will initiate the download of a pdf archive that contains the file. Oligonucleotide primers for RT-PCR amplification were designed and synthesized as described in Methods. Sequences are indicated for both forward and reverse primers used with either human ARPE-19 or chick primary RPE cells. The size of the predicted amplicon is indicated in base pairs.

## Appendix 2: Statistically over-represented TREs derived from the PAINT analysis of the human promoter regions.

To access the data, click or select the words “Appendix 2.” This will initiate the download of a pdf archive that contains the file. Represented for each TRE identified in the promoter sequences is the cluster in which the TRE was determined to be enriched, the TRANSFAC identifier and the p-value of the TRE in both gene sets.

## Appendix 3: Statistically over-represented TREs derived from the PAINT analysis of the human first intron regions.

To access the data, click or select the words “Appendix 3.” This will initiate the download of a pdf archive that contains the file. Represented for each TRE identified in the first intron sequences is the cluster in which the TRE was determined to be enriched, the TRANSFAC identifier and the p-value of the TRE in both gene sets.

## Appendix 4. Complete Candidate Interaction Matrix (CIM) for transcription response elements from PAINT analysis of human gene promoters.

To access the data, click or select the words “Appendix 4.” This will initiate the download of a pdf archive that contains the file. The RPE gene set was analyzed by PAINT and a graphic CIM was generated as described in Methods. The y-axis lists the Ensembl Gene identifiers for each gene and the x-axis lists the TRANSFAC TRE identifiers for each TRE found at least once in the promoter region. Genes listed along the y-axis are divided into two clusters that are either up-regulated (blue) or down-regulated (green) during EMT of RPE cells. TREs listed along the x-axis are clustered according to related occurrence pattern calculated using Jaccard's coefficient. The elements within the matrix are color-coded based upon the p-value of each TRE found in the regulatory regions of the genes. A red dot represents a TRE that is statistically significant and therefore over-represented in our gene set, while a blue dot signifies an under-represented TRE and a grey dot stands for a TRE with no statistical significance in our gene list. Note: This is a large-format figure and should be viewed at enhanced magnification to read the text in specific sections of the figure.

## Appendix 5. Complete Candidate Interaction Matrix (CIM) for transcription response elements from PAINT analysis of human gene first introns.

To access the data, click or select the words “Appendix 5.” This will initiate the download of a pdf archive that contains the file. The RPE gene set was analyzed by PAINT and a graphic CIM was generated as described in Methods. The y-axis lists the Ensembl Gene identifiers for each gene and the x-axis lists the TRANSFAC TRE identifiers for each TRE found at least once in the first intron region. Genes listed along the y-axis are divided into two clusters that are either up-regulated (blue) or down-regulated (green) during EMT of RPE cells. TREs listed along the x-axis are clustered according to related occurrence pattern calculated using Jaccard's coefficient. The elements within the matrix are color-coded based upon the p-value of each TRE found in the regulatory regions of the genes. A red dot represents a TRE that is statistically significant and therefore over-represented in our gene set, while a blue dot signifies an under-represented TRE and a grey dot stands for a TRE with no statistical significance in our gene list. Note: This is a large-format figure and should be viewed at enhanced magnification to read the text in specific sections of the figure.

## Appendix 6: Statistically over-represented TREs derived from the PAINT analysis of the mouse promoter regions.

To access the data, click or select the words “Appendix 6.” This will initiate the download of a pdf archive that contains the file. Represented for each TRE identified in the promoter sequences is the cluster in which the TRE was determined to be enriched, the TRANSFAC identifier and the p-value of the TRE in both gene sets.

## Appendix 7: Statistically over-represented TREs derived from the PAINT analysis of the mouse first intron regions.

To access the data, click or select the words “Appendix 7.” This will initiate the download of a pdf archive that contains the file. Represented for each TRE identified in the first intron sequences is the cluster in which the TRE was determined to be enriched, the TRANSFAC identifier and the p-value of the TRE in both gene sets.

## Appendix 8: Statistically over-represented TREs derived from the PAINT analysis of the rat promoter regions.

To access the data, click or select the words “Appendix 8.” This will initiate the download of a pdf archive that contains the file. Represented for each TRE identified in the promoter sequences is the cluster in which the TRE was determined to be enriched, the TRANSFAC identifier and the p-value of the TRE in both gene sets.

## Appendix 9: Statistically over-represented TREs derived from the PAINT analysis of the rat first intron regions.

To access the data, click or select the words “Appendix 9.” This will initiate the download of a pdf archive that contains the file. Represented for each TRE identified in the first intron sequences is the cluster in which the TRE was determined to be enriched, the TRANSFAC identifier and the p-value of the TRE in both gene sets.

## Appendix 10: Statistically over-represented TREs derived from the PAINT analysis of the chicken promoter regions.

To access the data, click or select the words “Appendix 10.” This will initiate the download of a pdf archive that contains the file. Represented for each TRE identified in the promoter sequences is the cluster in which the TRE was determined to be enriched, the TRANSFAC identifier and the p-value of the TRE in both gene sets.

## Appendix 11: Statistically over-represented TREs derived from the PAINT analysis of the chicken first intron regions.

To access the data, click or select the words “Appendix 11.” This will initiate the download of a pdf archive that contains the file. Represented for each TRE identified in the first intron sequences is the cluster in which the TRE was determined to be enriched, the TRANSFAC identifier and the p-value of the TRE in both gene sets.

## Appendix 12. Complete Candidate Interaction Matrix (CIM) for transcription response elements from PAINT analysis of mouse gene promoters.

To access the data, click or select the words “Appendix 12.” This will initiate the download of a pdf archive that contains the file. The RPE gene set was analyzed by PAINT and a graphic CIM was generated as described in Methods. The y-axis lists the Ensembl Gene identifiers for each gene and the x-axis lists the TRANSFAC TRE identifiers for each TRE found at least once in the promoter region. Genes listed along the y-axis are divided into two clusters that are either up-regulated (blue) or down-regulated (green) during EMT of RPE cells. TREs listed along the x-axis are clustered according to related occurrence pattern calculated using Jaccard's coefficient. The elements within the matrix are color-coded based upon the p-value of each TRE found in the regulatory regions of the genes. A red dot represents a TRE that is statistically significant and therefore over-represented in our gene set, while a blue dot signifies an under-represented TRE and a grey dot stands for a TRE with no statistical significance in our gene list. Note: This is a large-format figure and should be viewed at enhanced magnification to read the text in specific sections of the figure.

## Appendix 13. Complete Candidate Interaction Matrix (CIM) for transcription response elements from PAINT analysis of mouse gene first intron.

To access the data, click or select the words “Appendix 13.” This will initiate the download of a pdf archive that contains the file. The RPE gene set was analyzed by PAINT and a graphic CIM was generated as described in Methods. The y-axis lists the Ensembl Gene identifiers for each gene and the x-axis lists the TRANSFAC TRE identifiers for each TRE found at least once in the first intron region. Genes listed along the y-axis are divided into two clusters that are either up-regulated (blue) or down-regulated (green) during EMT of RPE cells. TREs listed along the x-axis are clustered according to related occurrence pattern calculated using Jaccard's coefficient. The elements within the matrix are color-coded based upon the p-value of each TRE found in the regulatory regions of the genes. A red dot represents a TRE that is statistically significant and therefore over-represented in our gene set, while a blue dot signifies an under-represented TRE and a grey dot stands for a TRE with no statistical significance in our gene list. Note: This is a large-format figure and should be viewed at enhanced magnification to read the text in specific sections of the figure.

## Appendix 14. Complete Candidate Interaction Matrix (CIM) for transcription response elements from PAINT analysis of rat gene promoters.

To access the data, click or select the words “Appendix 14.” This will initiate the download of a pdf archive that contains the file. The RPE gene set was analyzed by PAINT and a graphic CIM was generated as described in Methods. The y-axis lists the Ensembl Gene identifiers for each gene and the x-axis lists the TRANSFAC TRE identifiers for each TRE found at least once in the promoter region. Genes listed along the y-axis are divided into two clusters that are either up-regulated (blue) or down-regulated (green) during EMT of RPE cells. TREs listed along the x-axis are clustered according to related occurrence pattern calculated using Jaccard's coefficient. The elements within the matrix are color-coded based upon the p-value of each TRE found in the regulatory regions of the genes. A red dot represents a TRE that is statistically significant and therefore over-represented in our gene set, while a blue dot signifies an under-represented TRE and a grey dot stands for a TRE with no statistical significance in our gene list. Note: This is a large-format figure and should be viewed at enhanced magnification to read the text in specific sections of the figure.

## Appendix 15. Complete Candidate Interaction Matrix (CIM) for transcription response elements from PAINT analysis of rat gene first introns.

To access the data, click or select the words “Appendix 15.” This will initiate the download of a pdf archive that contains the file. The RPE gene set was analyzed by PAINT and a graphic CIM was generated as described in Methods. The y-axis lists the Ensembl Gene identifiers for each gene and the x-axis lists the TRANSFAC TRE identifiers for each TRE found at least once in the first introns region. Genes listed along the y-axis are divided into two clusters that are either up-regulated (blue) or down-regulated (green) during EMT of RPE cells. TREs listed along the x-axis are clustered according to related occurrence pattern calculated using Jaccard's coefficient. The elements within the matrix are color-coded based upon the p-value of each TRE found in the regulatory regions of the genes. A red dot represents a TRE that is statistically significant and therefore over-represented in our gene set, while a blue dot signifies an under-represented TRE and a grey dot stands for a TRE with no statistical significance in our gene list. Note: This is a large-format figure and should be viewed at enhanced magnification to read the text in specific sections of the figure.

## Appendix 16. Complete Candidate Interaction Matrix (CIM) for transcription response elements from PAINT analysis of chicken gene promoters.

To access the data, click or select the words “Appendix 16.” This will initiate the download of a pdf archive that contains the file. The RPE gene set was analyzed by PAINT and a graphic CIM was generated as described in Methods. The y-axis lists the Ensembl Gene identifiers for each gene and the x-axis lists the TRANSFAC TRE identifiers for each TRE found at least once in the promoter region. Genes listed along the y-axis are divided into two clusters that are either up-regulated (blue) or down-regulated (green) during EMT of RPE cells. TREs listed along the x-axis are clustered according to related occurrence pattern calculated using Jaccard's coefficient. The elements within the matrix are color-coded based upon the p-value of each TRE found in the regulatory regions of the genes. A red dot represents a TRE that is statistically significant and therefore over-represented in our gene set, while a blue dot signifies an under-represented TRE and a grey dot stands for a TRE with no statistical significance in our gene list. Note: This is a large-format figure and should be viewed at enhanced magnification to read the text in specific sections of the figure.

## Appendix 17. Complete Candidate Interaction Matrix (CIM) for transcription response elements from PAINT analysis of chicken gene first intron.

To access the data, click or select the words “Appendix 17.” This will initiate the download of a pdf archive that contains the file. The RPE gene set was analyzed by PAINT and a graphic CIM was generated as described in Methods. The y-axis lists the Ensembl Gene identifiers for each gene and the x-axis lists the TRANSFAC TRE identifiers for each TRE found at least once in the first intron region. Genes listed along the y-axis are divided into two clusters that are either up-regulated (blue) or down-regulated (green) during EMT of RPE cells. TREs listed along the x-axis are clustered according to related occurrence pattern calculated using Jaccard's coefficient. The elements within the matrix are color-coded based upon the p-value of each TRE found in the regulatory regions of the genes. A red dot represents a TRE that is statistically significant and therefore over-represented in our gene set, while a blue dot signifies an under-represented TRE and a grey dot stands for a TRE with no statistical significance in our gene list. Note: This is a large-format figure and should be viewed at enhanced magnification to read the text in specific sections of the figure.

## Appendix 18. Transcriptional regulatory network diagram for TREs associated with mouse promoter regions.

To access the data, click or select the words “Appendix 18.” This will initiate the download of a pdf archive that contains the file. The graphical representation was derived using GraphViz as described in Methods. The ellipses and diamonds represent individual genes divided into up-regulated (blue) and down-regulated (green) clusters. The boxes represent TREs, with arrows indicating gene-TRE associations. Note: This is a large-format figure and should be viewed at enhanced magnification to read the text in specific sections of the figure.

## Appendix 19. Transcriptional regulatory network diagram for TREs associated with rat promoter regions.

To access the data, click or select the words “Appendix 19.” This will initiate the download of a pdf archive that contains the file. The graphical representation was derived using GraphViz as described in Methods. The ellipses and diamonds represent individual genes divided into up-regulated (blue) and down-regulated (green) clusters. The boxes represent TREs, with arrows indicating gene-TRE associations. Note: This is a large-format figure and should be viewed at enhanced magnification to read the text in specific sections of the figure.

## Appendix 20. Transcriptional regulatory network diagram for TREs associated with chicken promoter regions.

To access the data, click or select the words “Appendix 20.” This will initiate the download of a pdf archive that contains the file. The graphical representation was derived using GraphViz as described in Methods. The ellipses and diamonds represent individual genes divided into up-regulated (blue) and down-regulated (green) clusters. The boxes represent TREs, with arrows indicating gene-TRE associations. Note: This is a large-format figure and should be viewed at enhanced magnification to read the text in specific sections of the figure.

## Appendix 21. Transcriptional regulatory network diagram for TREs associated with mouse first intron regions.

To access the data, click or select the words “Appendix 21.” This will initiate the download of a pdf archive that contains the file. The graphical representation was derived using GraphViz as described in Methods. The ellipses and diamonds represent individual genes divided into up-regulated (blue) and down-regulated (green) clusters. The boxes represent TREs, with arrows indicating gene-TRE associations. Note: This is a large-format figure and should be viewed at enhanced magnification to read the text in specific sections of the figure.

## Appendix 22. Transcriptional regulatory network diagram for TREs associated with rat first intron regions.

To access the data, click or select the words “Appendix 22.” This will initiate the download of a pdf archive that contains the file. The graphical representation was derived using GraphViz as described in Methods. The ellipses and diamonds represent individual genes divided into up-regulated (blue) and down-regulated (green) clusters. The boxes represent TREs, with arrows indicating gene-TRE associations. Note: This is a large-format figure and should be viewed at enhanced magnification to read the text in specific sections of the figure.

## Appendix 23. Transcriptional regulatory network diagram for TREs associated with chicken first intron regions.

To access the data, click or select the words “Appendix 23.” This will initiate the download of a pdf archive that contains the file. The graphical representation was derived using GraphViz as described in Methods. The ellipses and diamonds represent individual genes divided into up-regulated (blue) and down-regulated (green) clusters. The boxes represent TREs, with arrows indicating gene-TRE associations. Note: This is a large-format figure and should be viewed at enhanced magnification to read the text in specific sections of the figure.

## Appendix 24. Frequency analysis of TRE representation in mouse promoter regions.

To access the data, click or select the words “Appendix 24.” This will initiate the download of a pdf archive that contains the file. Frequency of occurrence of each TRE in the mouse gene promoter regions was determined from the PAINT analysis as described in Methods. The y-axis indicates the frequency of each TRE among the up-regulated (blue) or down-regulated (green) gene clusters as well as among the full background gene set (black).

## Appendix 25. Frequency analysis of TRE representation in mouse first intron regions.

To access the data, click or select the words “Appendix 25.” This will initiate the download of a pdf archive that contains the file. Frequency of occurrence of each TRE in the mouse gene first intron regions was determined from the PAINT analysis as described in Methods. The y-axis indicates the frequency of each TRE among the up-regulated (blue) or down-regulated (green) gene clusters as well as among the full background gene set (black).

## Appendix 26. Frequency analysis of TRE representation in rat promoter regions.

To access the data, click or select the words “Appendix 26.” This will initiate the download of a pdf archive that contains the file. Frequency of occurrence of each TRE in the rat gene promoter regions was determined from the PAINT analysis as described in Methods. The y-axis indicates the frequency of each TRE among the up-regulated (blue) or down-regulated (green) gene clusters as well as among the full background gene set (black).

## Appendix 27. Frequency analysis of TRE representation in rat first intron regions.

To access the data, click or select the words “Appendix 27.” This will initiate the download of a pdf archive that contains the file. Frequency of occurrence of each TRE in the rat gene first intron regions was determined from the PAINT analysis as described in Methods. The y-axis indicates the frequency of each TRE among the up-regulated (blue) or down-regulated (green) gene clusters as well as among the full background gene set (black).

## Appendix 28. Frequency analysis of TRE representation in chicken promoter regions.

To access the data, click or select the words “Appendix 28.” This will initiate the download of a pdf archive that contains the file. Frequency of occurrence of each TRE in the chicken gene promoter regions was determined from the PAINT analysis as described in Methods. The y-axis indicates the frequency of each TRE among the up-regulated (blue) or down-regulated (green) gene clusters as well as among the full background gene set (black).

## Appendix 29. Frequency analysis of TRE representation in chicken first intron regions.

To access the data, click or select the words “Appendix 29.” This will initiate the download of a pdf archive that contains the file. Frequency of occurrence of each TRE in the chicken gene first intron regions was determined from the PAINT analysis as described in Methods. The y-axis indicates the frequency of each TRE among the up-regulated (blue) or down-regulated (green) gene clusters as well as among the full background gene set (black).

## Appendix 30: Criteria table for transcription response element inclusion in gene promoter region regulatory network models.

To access the data, click or select the words “Appendix 30.” This will initiate the download of a pdf archive that contains the file. Represented for each TRE identified in the promoter regions of each gene are the TRANSFAC identifier name, the p-value, the ECF (evolutionary conservation factor), and the FR (frequency ratio) as defined in Methods. TREs included in this table are those with p-value <0.1, ECF =2, and FR >3.

## Appendix 31: Criteria table for transcription response element inclusion in gene first intron regulatory network models.

To access the data, click or select the words “Appendix 31.” This will initiate the download of a pdf archive that contains the file. Represented for each TRE identified in the first intron regions of each gene are the TRANSFAC identifier name, the p-value, the ECF (evolutionary conservation factor), and the FR (frequency ratio) as defined in Methods. TREs included in this table are those with p-value <0.1, ECF =2, and FR >3.

## References

[1] Martinez-Morales JR, Rodrigo I, Bovolenta P (2004). Eye development: a view from the retina pigmented epithelium.. Bioessays.

[2] Grunwald G. Structure and Function of the Retinal Pigment Epithelium.In: Tasman W, Jaeger EA, editors. Foundations of Clinical Ophthalmology. Philadelphia(PA): Lippincott, Williams and Wilkins; 2004. p. 1–21.

[3] Strauss O (2005). The retinal pigment epithelium in visual function.. Physiol Rev.

[4] Rodriguez de la Rua E, Pastor JC, Aragon J, Mayo-Iscar A, Martinez V, Garcia-Arumi J, Giraldo A, Sanabria-Ruiz Colmenares MR, Miranda I (2005). Interaction between surgical procedure for repairing retinal detachment and clinical risk factors for proliferative vitreoretinopathy.. Curr Eye Res.

[5] Grierson I, Hiscott P, Hogg P, Robey H, Mazure A, Larkin G (1994). Development, repair and regeneration of the retinal pigment epithelium.. Eye.

[6] Pastor JC (1998). Proliferative vitreoretinopathy: an overview.. Surv Ophthalmol.

[7] Thiery JP (2003). Epithelial-mesenchymal transitions in development and pathologies.. Curr Opin Cell Biol.

[8] Vincent-Salomon A, Thiery JP (2003). Host microenvironment in breast cancer development: epithelial-mesenchymal transition in breast cancer development.. Breast Cancer Res.

[9] Maeda M, Johnson KR, Wheelock MJ (2005). Cadherin switching: essential for behavioral but not morphological changes during an epithelium-to-mesenchyme transition.. J Cell Sci.

[10] Fan W, Zheng JJ, Peiper SC, McLaughlin BJ (2002). Changes in gene expression of ARPE-19 cells in response to vitreous treatment.. Ophthalmic Res.

[11] Singh S, Zheng JJ, Peiper SC, Mclaughlin BJ (2001). Gene expression profile of ARPE-19 during repair of the monolayer.. Graefes Arch Clin Exp Ophthalmol.

[12] Vadigepalli R, Chakravarthula P, Zak DE, Schwaber JS, Gonye GE (2003). PAINT: a promoter analysis and interaction network generation tool for gene regulatory network identification.. OMICS.

[13] Ashburner M, Ball CA, Blake JA, Botstein D, Butler H, Cherry JM, Davis AP, Dolinski K, Dwight SS, Eppig JT, Harris MA, Hill DP, Issel-Tarver L, Kasarskis A, Lewis S, Matese JC, Richardson JE, Ringwald M, Rubin GM, Sherlock G (2000). Gene ontology: tool for the unification of biology. The Gene Ontology Consortium.. Nat Genet.

[14] Hubbard T, Andrews D, Caccamo M, Cameron G, Chen Y, Clamp M, Clarke L, Coates G, Cox T, Cunningham F, Curwen V, Cutts T, Down T, Durbin R, Fernandez-Suarez XM, Gilbert J, Hammond M, Herrero J, Hotz H, Howe K, Iyer V, Jekosch K, Kahari A, Kasprzyk A, Keefe D, Keenan S, Kokocinsci F, London D, Longden I, McVicker G, Melsopp C, Meidl P, Potter S, Proctor G, Rae M, Rios D, Schuster M, Searle S, Severin J, Slater G, Smedley D, Smith J, Spooner W, Stabenau A, Stalker J, Storey R, Trevanion S, Ureta-Vidal A, Vogel J, White S, Woodwark C, Birney E (2005). Ensembl 2005.. Nucleic Acids Res.

[15] Hubbard T, Barker D, Birney E, Cameron G, Chen Y, Clark L, Cox T, Cuff J, Curwen V, Down T, Durbin R, Eyras E, Gilbert J, Hammond M, Huminiecki L, Kasprzyk A, Lehvaslaiho H, Lijnzaad P, Melsopp C, Mongin E, Pettett R, Pocock M, Potter S, Rust A, Schmidt E, Searle S, Slater G, Smith J, Spooner W, Stabenau A, Stalker J, Stupka E, Ureta-Vidal A, Vastrik I, Clamp M (2002). The Ensembl genome database project.. Nucleic Acids Res.

[16] Matys V, Fricke E, Geffers R, Gossling E, Haubrock M, Hehl R, Hornischer K, Karas D, Kel AE, Kel-Margoulis OV, Kloos DU, Land S, Lewicki-Potapov B, Michael H, Munch R, Reuter I, Rotert S, Saxel H, Scheer M, Thiele S, Wingender E (2003). TRANSFAC: transcriptional regulation, from patterns to profiles.. Nucleic Acids Res.

[17] Benjamini Y, Hochberg Y (1995). Controlling the false discovery rate: a practical and powerful approach to multiple testing.. J R Statist Soc B.

[18] Gansner ER, North SC (1999). An open graph visualization system and its applications to software engineering.. Softw Pract Exper.

[19] Hamburger V, Hamilton HL (1992). A series of normal stages in the development of the chick embryo. 1951.. Dev Dyn.

[20] Barishak YR (1992). Embryology of the eye and its adnexae.. Dev Ophthalmol.

[21] Vielkind U, Crawford BJ (1988). Evaluation of different procedures for the dissociation of retinal pigmented epithelium into single viable cells.. Pigment Cell Res.

[22] Dunn KC, Aotaki-Keen AE, Putkey FR, Hjelmeland LM (1996). ARPE-19, a human retinal pigment epithelial cell line with differentiated properties.. Exp Eye Res.

[23] Philp NJ, Wang D, Yoon H, Hjelmeland LM (2003). Polarized expression of monocarboxylate transporters in human retinal pigment epithelium and ARPE-19 cells.. Invest Ophthalmol Vis Sci.

[24] Giegerich R, Meyer F, Schleiermacher C (1996). GeneFisher–software support for the detection of postulated genes.. Proc Int Conf Intell Syst Mol Biol.

[25] Altschul SF, Madden TL, Schaffer AA, Zhang J, Zhang Z, Miller W, Lipman DJ (1997). Gapped BLAST and PSI-BLAST: a new generation of protein database search programs.. Nucleic Acids Res.

[26] Grunwald GB (1996). Cadherin cell adhesion molecules in retinal development and pathology.. Progress in Retinal and Eye Research.

[27] Zhao S, Rizzolo LJ, Barnstable CJ (1997). Differentiation and transdifferentiation of the retinal pigment epithelium.. Int Rev Cytol.

[28] Murray SA, Gridley T (2006). Snail family genes are required for left-right asymmetry determination, but not neural crest formation, in mice.. Proc Natl Acad Sci USA.

[29] Come C, Magnino F, Bibeau F, De Santa Barbara P, Becker KF, Theillet C, Savagner P (2006). Snail and slug play distinct roles during breast carcinoma progression.. Clin Cancer Res.

[30] Addya S, Keller MA, Delgrosso K, Ponte CM, Vadigepalli R, Gonye GE, Surrey S (2004). Erythroid-induced commitment of K562 cells results in clusters of differentially expressed genes enriched for specific transcription regulatory elements.. Physiol Genomics.

[31] Merika M, Orkin SH (1993). DNA-binding specificity of GATA family transcription factors.. Mol Cell Biol.

[32] Crossley M, Orkin SH (1994). Phosphorylation of the erythroid transcription factor GATA-1.. J Biol Chem.

[33] Tronche F, Yaniv M (1992). HNF1, a homeoprotein member of the hepatic transcription regulatory network.. Bioessays.

[34] von Strandmann EP, Nastos A, Holewa B, Senkel S, Weber H, Ryffel GU (1997). Patterning the expression of a tissue-specific transcription factor in embryogenesis: HNF1 alpha gene activation during Xenopus development.. Mech Dev.

[35] Haumaitre C, Reber M, Cereghini S (2003). Functions of HNF1 family members in differentiation of the visceral endoderm cell lineage.. J Biol Chem.

[36] Kemler I, Schaffner W (1990). Octamer transcription factors and the cell type-specificity of immunoglobulin gene expression.. FASEB J.

[37] Prescott JL, Blok L, Tindall DJ (1998). Isolation and androgen regulation of the human homeobox cDNA, NKX3.1.. Prostate.

[38] Ueyama T, Kasahara H, Ishiwata T, Nie Q, Izumo S (2003). Myocardin expression is regulated by Nkx2.5, and its function is required for cardiomyogenesis.. Mol Cell Biol.

[39] Akazawa H, Komuro I (2005). Cardiac transcription factor Csx/Nkx2–5: Its role in cardiac development and diseases.. Pharmacol Ther.

[40] Korkmaz KS, Korkmaz CG, Ragnhildstveit E, Kizildag S, Pretlow TG, Saatcioglu F (2000). Full-length cDNA sequence and genomic organization of human NKX3A-alternative forms and regulation by both androgens and estrogens.. Gene.

[41] He WW, Sciavolino PJ, Wing J, Augustus M, Hudson P, Meissner PS, Curtis RT, Shell BK, Bostwick DG, Tindall DJ, Gelmann EP, Abate-Shen C, Carter KC (1997). A novel human prostate-specific, androgen-regulated homeobox gene (NKX3.1) that maps to 8p21, a region frequently deleted in prostate cancer.. Genomics.

[42] Mamane Y, Heylbroeck C, Genin P, Algarte M, Servant MJ, LePage C, DeLuca C, Kwon H, Lin R, Hiscott J (1999). Interferon regulatory factors: the next generation.. Gene.

[43] Romeo G, Fiorucci G, Chiantore MV, Percario ZA, Vannucchi S, Affabris E (2002). IRF-1 as a negative regulator of cell proliferation.. J Interferon Cytokine Res.

[44] Shimano H (2000). Sterol regulatory element-binding protein-1 as a dominant transcription factor for gene regulation of lipogenic enzymes in the liver.. Trends Cardiovasc Med.

[45] Rajendran JG, Mankoff DA, O'Sullivan F, Peterson LM, Schwartz DL, Conrad EU, Spence AM, Muzi M, Farwell DG, Krohn KA (2004). Hypoxia and glucose metabolism in malignant tumors: evaluation by [18F]fluoromisonidazole and [18F]fluorodeoxyglucose positron emission tomography imaging.. Clin Cancer Res.

[46] Blyth K, Cameron ER, Neil JC (2005). The RUNX genes: gain or loss of function in cancer.. Nat Rev Cancer.

[47] Kuiper RP, Schepens M, Thijssen J, Schoenmakers EF, van Kessel AG (2004). Regulation of the MiTF/TFE bHLH-LZ transcription factors through restricted spatial expression and alternative splicing of functional domains.. Nucleic Acids Res.

[48] Hua X, Miller ZA, Benchabane H, Wrana JL, Lodish HF (2000). Synergism between transcription factors TFE3 and Smad3 in transforming growth factor-beta-induced transcription of the Smad7 gene.. J Biol Chem.

[49] Verastegui C, Bertolotto C, Bille K, Abbe P, Ortonne JP, Ballotti R (2000). TFE3, a transcription factor homologous to microphthalmia, is a potential transcriptional activator of tyrosinase and TyrpI genes.. Mol Endocrinol.

[50] Datto MB, Frederick JP, Pan L, Borton AJ, Zhuang Y, Wang XF (1999). Targeted disruption of Smad3 reveals an essential role in transforming growth factor beta-mediated signal transduction.. Mol Cell Biol.

[51] Phanish MK, Wahab NA, Colville-Nash P, Hendry BM, Dockrell ME (2006). The differential role of Smad2 and Smad3 in the regulation of pro-fibrotic TGFbeta1 responses in human proximal-tubule epithelial cells.. Biochem J.

[52] Valcourt U, Kowanetz M, Niimi H, Heldin CH, Moustakas A (2005). TGF-beta and the Smad signaling pathway support transcriptomic reprogramming during epithelial-mesenchymal cell transition.. Mol Biol Cell.

[53] Sato M, Muragaki Y, Saika S, Roberts AB, Ooshima A (2003). Targeted disruption of TGF-beta1/Smad3 signaling protects against renal tubulointerstitial fibrosis induced by unilateral ureteral obstruction.. J Clin Invest.

[54] Hua X, Liu X, Ansari DO, Lodish HF (1998). Synergistic cooperation of TFE3 and smad proteins in TGF-beta-induced transcription of the plasminogen activator inhibitor-1 gene.. Genes Dev.

[55] Zavadil J, Bottinger EP (2005). TGF-beta and epithelial-to-mesenchymal transitions.. Oncogene.

[56] Nijman SM, Hijmans EM, El Messaoudi S, van Dongen MM, Sardet C, Bernards R (2006). A functional genetic screen identifies TFE3 as a gene that confers resistance to the anti-proliferative effects of the retinoblastoma protein and transforming growth factor-beta.. J Biol Chem.

[57] Saika S, Kono-Saika S, Tanaka T, Yamanaka O, Ohnishi Y, Sato M, Muragaki Y, Ooshima A, Yoo J, Flanders KC, Roberts AB (2004). Smad3 is required for dedifferentiation of retinal pigment epithelium following retinal detachment in mice.. Lab Invest.

[58] LaGamba D, Nawshad A, Hay ED (2005). Microarray analysis of gene expression during epithelial-mesenchymal transformation.. Dev Dyn.

[59] Bindels S, Mestdagt M, Vandewalle C, Jacobs N, Volders L, Noel A, van Roy F, Berx G, Foidart JM, Gilles C (2006). Regulation of vimentin by SIP1 in human epithelial breast tumor cells.. Oncogene.

[60] Kang Y, Massague J (2004). Epithelial-mesenchymal transitions: twist in development and metastasis.. Cell.

[61] Martinez-Estrada OM, Culleres A, Soriano FX, Peinado H, Bolos V, Martinez FO, Reina M, Cano A, Fabre M, Vilaro S (2006). The transcription factors Slug and Snail act as repressors of Claudin-1 expression in epithelial cells.. Biochem J.

[62] Hovanes K, Li TW, Waterman ML (2000). The human LEF-1 gene contains a promoter preferentially active in lymphocytes and encodes multiple isoforms derived from alternative splicing.. Nucleic Acids Res.

[63] Tian J, Ishibashi K, Handa JT (2004). The expression of native and cultured RPE grown on different matrices.. Physiol Genomics.

[64] Tian J, Ishibashi K, Honda S, Boylan SA, Hjelmeland LM, Handa JT (2005). The expression of native and cultured human retinal pigment epithelial cells grown in different culture conditions.. Br J Ophthalmol.

